# Transcriptome Analysis of Wnt3a-Treated Triple-Negative Breast Cancer Cells

**DOI:** 10.1371/journal.pone.0122333

**Published:** 2015-04-07

**Authors:** Sylvie Maubant, Bruno Tesson, Virginie Maire, Mengliang Ye, Guillem Rigaill, David Gentien, Francisco Cruzalegui, Gordon C. Tucker, Sergio Roman-Roman, Thierry Dubois

**Affiliations:** 1 Breast Cancer Biology Group, Translational Research Department, Institut Curie, Centre de Recherche, Paris, France; 2 INSERM U900, Bioinformatics, Biostatistics, Epidemiology and Computational Systems Biology of Cancer, Institut Curie, Centre de Recherche, Paris, France; 3 Mines ParisTech, Fontainebleau, France; 4 Unité de Recherche en Génomique Végétale, INRA-CNRS-Université d'Evry Val d'Essonne, Evry, France; 5 Platform of Molecular Biology Facilities, Translational Research Department, Institut Curie, Centre de Recherche, Paris, France; 6 Institut de Recherches SERVIER, Pôle Innovation Thérapeutique Oncologie, Croissy-sur-Seine, France; 7 Translational Research Department, Institut Curie, Centre de Recherche, Paris, France

## Abstract

The canonical Wnt/β-catenin pathway is activated in triple-negative breast cancer (TNBC). The activation of this pathway leads to the expression of specific target genes depending on the cell/tissue context. Here, we analyzed the transcriptome of two different TNBC cell lines to define a comprehensive list of Wnt target genes. The treatment of cells with Wnt3a for 6h up-regulated the expression (fold change > 1.3) of 59 genes in MDA-MB-468 cells and 241 genes in HCC38 cells. Thirty genes were common to both cell lines. Beta-catenin may also be a transcriptional repressor and we found that 18 and 166 genes were down-regulated in response to Wnt3a treatment for 6h in MDA-MB-468 and HCC38 cells, respectively, of which six were common to both cell lines. Only half of the activated and the repressed transcripts have been previously described as Wnt target genes. Therefore, our study reveals 137 novel genes that may be positively regulated by Wnt3a and 104 novel genes that may be negatively regulated by Wnt3a. These genes are involved in the Wnt pathway itself, and also in TGFβ, p53 and Hedgehog pathways. Thorough characterization of these novel potential Wnt target genes may reveal new regulators of the canonical Wnt pathway. The comparison of our list of Wnt target genes with those published in other cellular contexts confirms the notion that Wnt target genes are tissue-, cell line- and treatment-specific. Genes up-regulated in Wnt3a-stimulated cell lines were more strongly expressed in TNBC than in luminal A breast cancer samples. These genes were also overexpressed, but to a much lesser extent, in HER2+ and luminal B tumors. We identified 72 Wnt target genes higher expressed in TNBCs (17 with a fold change >1.3) which may reflect the chronic activation of the canonical Wnt pathway that occurs in TNBC tumors.

## Introduction

Breast cancer is one of the most common tumors in women. It is a complex, heterogeneous disease comprising several subgroups of pathologies with different patient outcomes [1–3]. Triple-negative breast cancer (TNBC), closely related to basal-like breast cancer (BLBC), is characterized by an absence of estrogen receptor (ER) and progesterone receptor (PR) expression and a lack of human epidermal growth factor receptor 2 (HER2) overexpression/amplification. TNBC itself constitutes a heterogeneous group of breast cancer [4–6], which is highly proliferative and genetically instable, and associated with a poor prognosis. Unlike other breast cancer subtypes, such as luminal (expressing ER and PR) and HER2-overexpressing (HER2+) tumors, TNBC cannot be treated with targeted therapies, such as tamoxifen or anti-HER2 antibodies. TNBC patients are therefore treated exclusively with conventional cytotoxic therapies, but about half of them present relapse and metastasis within the first three to five years after treatment [7]. Therefore, treatment of patients with TNBC remains a major challenge for oncologists and alternative treatments to conventional chemotherapies are needed to improve their survivals.

The Wnt signaling pathway mediates biological processes such as cell adhesion, migration, proliferation, differentiation and survival [8–10]. It consists of two main arms: the canonical (Wnt/β-catenin) and the non-canonical pathways, which differ in terms of their dependence on β-catenin [11,12]. The activation of the canonical Wnt pathway leads to the stabilization of β-catenin which translocates to the nucleus and induces the expression of Wnt target genes. Besides its function in normal cells/tissues, Wnt signaling can become deregulated during human disease. The best documented example is the tumorigenesis of colorectal cancer [13]. The Wnt/β-catenin pathway is also activated in human breast cancer, in particular in the TNBC/BLBC breast cancer subtype that is associated with poor prognosis [14]. Indeed, the activated form of β-catenin has been observed in breast cancer [15–18], and is frequently found in the TNBC/BLBC subtype [14,19–23]. The aberrant activation of the Wnt/β-catenin pathway in mice leads to mammary carcinogenesis [24], and transgenic mice expressing a constitutively active form of β-catenin in the mammary gland develop basal-like tumors [25], suggesting a crucial role for the canonical Wnt pathway in TNBC/BLBC tumorigenesis. Mutations of genes encoding intracellular components of the canonical pathway, including *APC* (encoding adenomatous polyposis coli), *CTNNB1* (encoding β-catenin) and *AXIN*, are frequent in colorectal and hepatocellular cancers [11,12,26], but are rare in breast cancer [22–24,27–30]. Instead, deregulated expression of cell surface components such as LRP6 or FZD7 transmembrane receptors may be responsible for the activation of the Wnt pathway in TNBC/BLBC [14,19–23].

Nuclear localization of β-catenin and the expression of Wnt target genes reflect the activation of the Wnt/β-catenin pathway. Nevertheless, the detection of nuclear β-catenin is experimentally challenging and dependent on the tissues and the cell lines tested. An alternative way to evaluate the activation of the canonical Wnt pathway is to measure the expression of Wnt/β-catenin target genes. However, Wnt target genes vary substantially depending on the cellular/tissue context. The exceptions are *AXIN2* and *NKD1* which are considered as universal Wnt target genes [12]. The role of β-catenin in the transcriptional activation of its target genes is well documented. However, recent studies have also reported a link between β-catenin and transcriptional repression, which is an underestimated aspect of the Wnt signaling [31–33]. Several methodological approaches have been undertaken to identify Wnt target genes in different cellular or tissue contexts: stimulation of cells with Wnt3a or Wnt1 ligand (recombinant protein or plasmid) [34,35]; depletion of β-catenin (siRNA) [36] or Tcf (dominant negative construct) [37]; overexpression of active β-catenin (plasmid) [36]; evaluation of Wnt signaling activity (nuclear staining of β-catenin [38,39], mutations of the *CTNNB1* gene [40]); and screening to identify binding sites for the Tcf transcription factor in DNA sequences [34]. Wnt target genes have been mostly examined in colon and also in ovarian and liver cancers [37–39], but are not frequently examined in breast cancer. Some Wnt target genes are components of the Wnt pathway itself. Such targets are mostly inhibitors (e.g., AXIN2 and NKD1) and probably prevent the uncontrolled activation of the pathway through negative feedback loops. Therefore, the characterization of β-catenin target genes in breast tissue may lead to the discovery of new regulators of the Wnt pathway and improve our understanding of TNBC tumorigenesis. We thus used microarrays to investigate the expression of 19,738 transcripts following Wnt3a stimulation in two TNBC cell lines, HCC38 and MDA-MB-468, and we report a comprehensive list of genes that are activated or repressed in breast cancer by Wnt3a. Pathway analysis revealed that the Wnt target genes were mainly associated with the Wnt, TGFβ, p53 and Hedgehog pathways. The comparison of our list of Wnt target genes with those previously identified in fibroblast and epithelial cell lines confirms that the identity of Wnt target genes is highly dependent on the cellular/tissue context. We examined the expression level of our lists of Wnt target genes in 130 human breast tumors. We found that 72 Wnt target genes (17 with a fold change > 1.3) may reflect the activation of the canonical Wnt pathway in a more chronic situation.

## Materials and Methods

### Triple-negative cell lines

BT20, BT549, HCC38, HCC70, HCC1187, HCC1937, MDA-MB-157, MDA-MB-231 and MDA-MB-468 cells were purchased in May 2006 and May 2008 from the American Type Culture Collection (LGC Standards, Molsheim, France). Cells were characterized by DNA and RNA microarrays [41,42] and authenticated in 2013 by short tandem repeat profiling (data not shown).

BT20 cells were cultured in MEM(Eagle) (Sigma, Saint Quentin Fallavier, France) containing 1% Glutamax (Invitrogen), 10% fetal bovine serum (FBS, Invitrogen, Cergy Pontoise, France), 1.5g/L sodium bicarbonate (Invitrogen), 0.1mM non-essential amino-acids (Invitrogen) and 1mM sodium pyruvate (Invitrogen). BT549 and MDA-MB-468 cells were maintained in RPMI-1640 containing Glutamax (Invitrogen) and supplemented with 10% FBS. HCC38, HCC70, HCC1187, HCC1937 cells were cultured in RPMI-1640 with Glutamax containing 10% FBS, 1.5g/L sodium bicarbonate, 10mM Hepes (Invitrogen) and 1mM sodium pyruvate. MDA-MB-157 cells were maintained in Leibovitz's L-15 medium containing Glutamax (Invitrogen) and supplemented with 10% FBS and 10mM Hepes. MDA-MB-231 cells were cultured in DMEM-F12 with Glutamax (Invitrogen) containing 10% FBS. Antibiotics were added to all media (100U/mL penicillin and 100μg/mL streptomycin). Cells were cultured at 37°C in a 5% CO_2_ humidified incubator. For all experiments, cells were used up to the 15th passage after thawing.

### Transfection of plasmid DNA

The Wnt-responsive element-luciferase reporter WRE was kindly provided by the Galapagos company (Romainville, France), the mutant-responsive element-luciferase variant MRE by Ron Smits (Rotterdam, The Netherlands) [43] and the pRL-TK plasmid by the Servier company (Croissy-sur-Seine, France). The construct pRK5-SK-β-cateninΔGSK and empty vector were obtained from René Bernards (Ultrecht, The Netherlands) [44] and Maria Carla Parrini (Institut Curie Paris, France), respectively.

FuGENE HD reagent (Promega, Charbonnières-les-Bains, France) was used to transiently transfect cells with plasmids according to the manufacturer’s recommendations.

### Compounds

Recombinant human Wnt3a (R&D Systems, Lille, France) was reconstituted at 10μg/mL in PBS containing 0.1% BSA, then used in experiments at a final concentration of 100ng/mL. This concentration of Wnt3a is routinely used to activate the Wnt pathway in mammary cells, in particular in TNBC cell lines [35,45–48]. We have shown that this concentration of Wnt3a leads to the activation of the Wnt signaling pathway (S1 Fig).

### Antibodies

Primary antibodies used were mouse monoclonal anti-β-catenin (clone 14/beta-catenin, BD Transduction Laboratories, Le Pont de Claix, France; 1:1,000) or anti-active-β-catenin (clone 8E7, Millipore, Molsheim, France; 1:500), rabbit monoclonal anti-LRP5 (clone D5G4, Cell Signaling Technology, Ozyme, Saint Quentin en Yvelines, France; 1:1,000), anti-LRP6 (clone C5C7, Cell Signaling Technology; 1:500), and mouse monoclonal anti-actin (clone AC-15, Sigma; 1:5,000). The secondary antibodies used were horseradish peroxidase-conjugated anti-mouse or anti-rabbit IgG (Jackson ImmunoResearch, Interchim, Montluçon, France; 1:20,000).

### SDS-PAGE and western blotting

Cells were lysed in Laemmli buffer containing 50mM Tris (pH 6.8), 2% sodium dodecyl sulfate (SDS), 5% glycerol, 2mM 1,4-dithio-DL-threitol, 2.5mM ethylenediaminetetraacetic acid, 2.5mM ethylene glycol tetraacetic acid, 2mM sodium orthovanadate, 10mM sodium fluoride and a cocktail of protease (Roche) and phosphatase (Pierce, Perbio, Brebières, France) inhibitors. Protein concentration in each sample was determined with the reducing agent compatible version of the BCA Protein Assay kit (Pierce). Equal amounts of total protein were fractionated under reducing conditions by SDS–PAGE and then blotted onto PVDF membranes (Bio-Rad, Marnes-la-Coquette, France). The membranes were blocked with 5% BSA or 10% skimmed milk in TBS containing 0.1% Tween 20 (TBS-T), hybridized with the primary antibody of interest overnight at 4°C. Membranes were washed in TBS-T and then hybridized with the secondary antibody for one hour at room temperature. Antibodies were diluted in TBS-T containing 5% BSA or 10% skimmed milk. After washing with TBS-T, immune complexes on membranes were detected by enhanced chemiluminescence (Amersham, GE Healthcare, Orsay, France). Actin was used as loading control.

### Beta-catenin reporter assay

Cells were transiently transfected with either the reporter plasmid WRE (containing Tcf-binding sites driving the transcription of the firefly luciferase enzyme) or the mutant variant MRE (containing inactive Tcf-binding sites) as described above. Co-transfection with pRL-TK, which encodes a Renilla luciferase gene downstream from a minimal HSV-TK promoter, was systematically performed to normalize for transfection efficiency. Eight hours after transfection, cells were washed and cultured overnight in culture medium without serum, then Wnt3a was added for 3, 6, 9 or 12 hours.

For experiments in which the construct pRK5-SK-β-cateninΔGSK or pRK5-SK was used, cells were co-transfected with WRE and pRL-TK or MRE and pRL-TK. Under these conditions, cells were cultured for 6, 12 or 24 hours.

After treatment (Wnt3a stimulation or overexpression of active β-catenin), cells were lysed, and a luciferase assay was performed with the Dual-Luciferase Reporter Assay Kit (Promega), according to the manufacturer's instructions.

Triplicates for each condition were included in the experiment and the experiment was repeated at least twice.

### Microarray analysis in cell lines

Cells were seeded in six-well plates, serum starved overnight, and then treated with Wnt3a for the indicated times (6, 12 or 24 hours). Triplicates for each condition were included in the experiment. Total RNA was extracted with the RNeasy Mini Kit from Qiagen (Courtaboeuf, France) following the manufacturer’s recommendations. After RNA quality and quantity controls, samples were hybridized onto Gene st 1.1 Affymetrix chips. Samples were processed as described on the website of the company. The data were analyzed with the brainarray HuGene11stv1_Hs_ENTREZG version 14 custom chipset definition file for the HuGene11stv1 affymetrix array [49]. The data were first log2 transformed and normalized with RMA [50]. A linear model was fitted with limma [51] including three factors that were treated as fixed effects: cell line (either HCC38 or MDA-MB-468), time (6, 12 or 24 hours) and treatment (Wnt3a stimulated or control) and all possible interaction terms. For each cell line at each time point, the significance of differences between Wnt3a-treated and control cells was determined and a correction for multiple testing was applied with Benjamini & Hochberg’s methodology [52]. Only genes with significantly different expression (*P* < 0.05), with a log2 fold change superior to 0.3785 (i.e. fold change > 1.3) or inferior to -0.3785 (i.e. fold change < 1.3), were selected. Pathway enrichment analysis was carried out with GeneTrail [53]. A hypergeometric test, corrected for multiple testing with the Benjamini-Hochberg method, was used to assess the significance of the over-representation of biological annotations among gene lists. The transcriptomic data of Wnt3a-treated cells are available in Gene Expression Omnibus (GEO) (accession number: GSE65238).

### Quantitative real time reverse transcription-polymerase chain reaction (qRT-PCR)

Cells were seeded in six-well plates, serum starved overnight, and then treated with Wnt3a for 3, 6, 9 or 12 hours. For experiments in which cells were transfected with the construct pRK5-SK-β-cateninΔGSK or the empty vector pRK5-SK, cells were cultured for 12, 24 or 48 hours. Triplicates for each condition were included in the experiment and the experiment was repeated at least three times.

Total RNA was extracted with the RNeasy Mini Kit (Qiagen). For each reaction, 50 to 100ng RNA was combined with components of KAPA SYBR FAST One-Step qRT-PCR Kit (KapaBiosystems, Clinisciences, Nanterre, France) and the Quantitect primers (Qiagen) to a final volume of 20μL, according to instructions of the manufacturers. The reaction mix was subjected to qRT-PCR performed with the 7900HT apparatus and SDS2.4 software (Applied Biosystems) with the following settings: 5 min at 42°C (RT step), 5 min incubation at 95°C followed by a three-step cycling program with 40 cycles of 15 sec at 95°C, 30 sec at 60°C and 30 sec at 72°C (PCR step). A post-PCR dissociation analysis step was included according to instrument guidelines to distinguish specific from non-specific amplification products. All data were normalized to endogenous actin expression.

### Tissue samples and microarray data

The human samples used in this study have been previously described [41,42]. RNA microarray (Affymetrix U133 Plus 2.0) performed on 41 TNBC, 30 HER2^+^, 30 luminal B (LB), 29 luminal A (LA) and 11 healthy tissue breast samples, have also been previously described [41,42] (GEO accession number: GSE65216).

### Analysis of the enrichment of the up- and down-regulated Wnt target genes in human breast tumor samples

We analyzed whether the Wnt target genes we identified were enriched in human tumor samples. We compared the gene expression data obtained in TNBC cell lines stimulated with Wnt3a with that obtained in our cohort of 130 human breast cancer samples [41,42]. The two experiments were not done with the same arrays: the cell line (Gene st 1.1, Affymetrix) and the tumor (U133 Plus 2.0, Affymetrix) experiments contained 19738 and 11543 genes, respectively. We restricted our study to the 11262 genes present on both arrays. Of note, *AXIN2* was not found in our tumor dataset. We applied a FDR (False Discovery Rate) cut-off of 0.05 and a log-2 fold change threshold of 0.

We used the Fisher exact test to assess the significance of the intersection between the genes that were up- or down-regulated upon the stimulation of cell lines with Wnt3a, and those that were more strongly or more poorly expressed in tumors (TNBC, HER2+, LB) than in LA samples. Several gene sets were considered. For the cell line experiment, we considered the up- or down-regulated genes, the different time points (6, 12 and 24 hours) and the two TNBC cell lines. For the breast tumors, we considered the genes that were more strongly or more poorly expressed in the three different subgroups of tumors (TNBC, HER2+ and LB) than in LA samples. Each subgroup was considered separately and all comparisons between the different breast cancer subtypes were performed.

The comparisons between tumors and cell lines were performed with data obtained in HCC38 and MDA-MB-468 cell lines. However, there was no significant overlap between the MDA-MB-468 and the tumor datasets, possibly because of the very low number of genes found to be differentially expressed in Wnt3a-stimulated MDA-MB-468 cells. Therefore, we focused only on the data obtained with HCC38 cells.

The gene lists were as follows:

MDA-MB-468 cells: 6h up-regulated (66 genes), 12h up-regulated (74 genes), 24h up-regulated (20 genes), 6h down-regulated (34 genes), 12h down-regulated (43 genes), 24h down-regulated (12 genes).
HCC38 cells: 6h up-regulated (419 genes), 12h up-regulated (907 genes), 24h up-regulated (653 genes), 6h down-regulated (256 genes), 12h down-regulated (707 genes), 24h down-regulated (530 genes).
Tumor samples (relative to LA): TNBC up-regulated (3497 genes), HER2+ up-regulated (2110 genes), LB up-regulated (1587 genes), TNBC down-regulated (2971 genes), HER2+ down-regulated (2001 genes), LB down-regulated (1184 genes).
Other lists of genes up- or down-regulated in tumors: TNBC vs HER2+ (up-regulated: 2242 genes; down-regulated: 1865 genes), TNBC vs LB (up-regulated: 2775 genes; down-regulated: 2725 genes), HER2+ vs LB (up-regulated: 581 genes; down-regulated: 581 genes).

In addition, we selected the Wnt target genes that were up-regulated at both the earliest (6h) and the latest time (24h) point after Wnt3a stimulation, to identify potential up-regulated Wnt target genes that could reflect the chronic activation of the Wnt pathway in human cancer. The analysis was performed with the 133 Wnt target genes up-regulated in HCC38 cells, which displayed a higher number of up-regulated genes than in MDA-MB-468 cells (only 13 genes were up-regulated at both 6h and 24h). Of these 133 genes that were up-regulated in HCC38 cells at both 6h and 24h after Wnt3a stimulation, 72 were more strongly expressed in TNBC than in LA tumors. We applied a fold change of > 1.3, which was defined for the cell line experiment, to select the most differently expressed Wnt target genes. Seventeen out of 72 genes were more strongly expressed in TNBC than in LA tumors, at this fold change. We generated a heatmap of the genes ordered by their *P* value in the t-test.

We used the Fisher exact test to assess the significance of the overlap between two gene lists. For all analyses comparing cell lines and tumors, we used an adjusted (Benjamini Hochberg) *P* value cut off of 5% and no fold change threshold.

### Statistical analyses for *in vitro* experiments

Data are presented as the mean ± standard deviation (SD). Differences between groups were determined with Student's t-test. Results were considered significant at a *P* value lower than 0.05.

## Results and Discussion

### The canonical Wnt pathway is activated in triple-negative breast cancer cell lines

**Fig 1:**
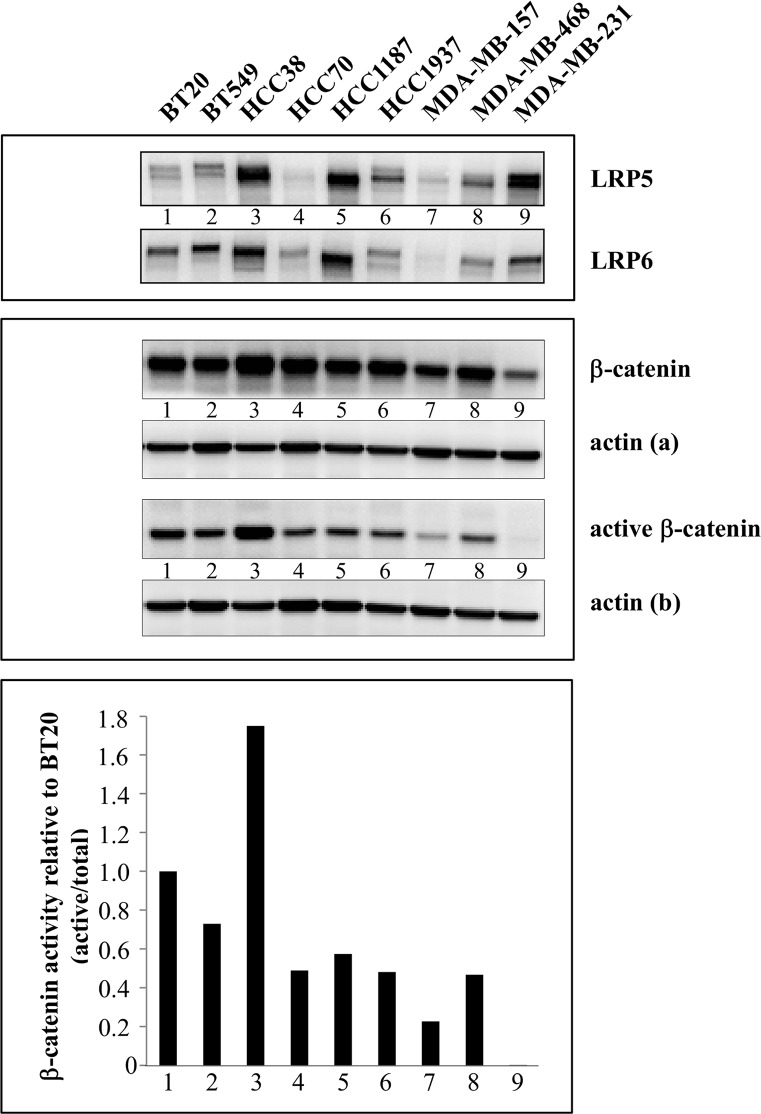
Expression of β-catenin, LRP5 and LRP5 in TNBC cell lines. The abundance of LRP5, LRP6 and β-catenin (total and active forms) was evaluated in different TNBC cell lines by western blotting. Actin was used as a loading control: actin (a) for LRP5 and beta-catenin; actin (b) for LRP6 and active beta-catenin.

We sought to evaluate Wnt activity in TNBC cell lines; therefore, we measured the abundance of total β-catenin and its unphosphorylated, active form in TNBC cell lines under resting conditions (Fig 1). The abundance of the total and active forms of β-catenin varied in the different cell lines (Fig 1). Except for MDA-MB-231, the canonical Wnt pathway was active in all tested TNBC cell lines in unstimulated conditions, and was most active in HCC38 cells (Fig 1).

We examined whether Wnt3a, a Wnt ligand commonly used to stimulate the canonical Wnt pathway, could stimulate Wnt/β-catenin signaling beyond basal levels observed in HCC38 and MDA-MB-468 cell lines, which displayed differences in their basal Wnt activity (Fig 1). Tcf-mediated transcriptional activity was measured with a reporter plasmid that expresses the luciferase gene under the control of several Tcf binding sites (see Material and Methods section). For both cell lines, Wnt/β-catenin activity was higher than in control cells as early as 3h following the incubation of cells with Wnt3a (S1 Fig). This activation was highest 6h post-treatment and started to decrease at 12h (S1 Fig), indicating that the transcriptional activity of β-catenin is optimal 6 hours following the initial stimulus. Although the transfection of HCC38 cells was not optimal (not shown), luciferase activity was higher in HCC38 cells than in MDA-MB-468 cells (S1 Fig), indicating that Wnt3a activates the Wnt pathway more strongly in HCC38 cells than in MDA-MB-468 cells. This may be because the Wnt3a receptors, LRP5 and LRP6, are more strongly expressed in HCC38 cells than in MDA-MB-468 cells (Fig 1).

Altogether, these results indicate that the canonical Wnt pathway is active in TNBC cell lines, and that Wnt3a further stimulates it.

### Genes activated by Wnt3a in HCC38 and MDA-MB-468 triple-negative breast cancer cell lines

**Fig 2:**
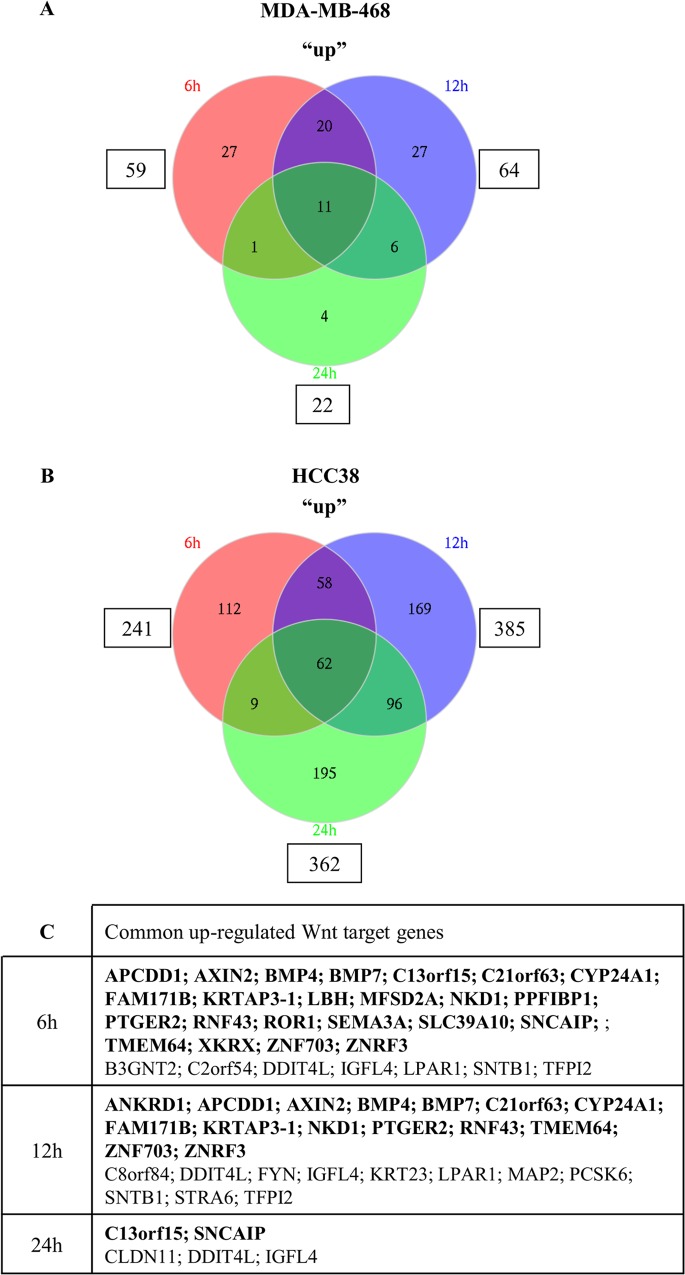
Wnt3a-dependent gene activation in MDA-MB-468 and HCC38 cells. Venn diagrams indicate the number of genes that were up-regulated in MDA-MB-468 (A) or HCC38 cells (B) following Wnt3a stimulation for 6, 12 or 24 hours. Genes found in common in both cell lines are listed in the table and known Wnt target genes are shown in bold (C).

The expression of Wnt target genes appears to be cell/tissue context dependent [31,32]. We therefore performed expression profiling with Gene st 1.1 Affymetrix chips in HCC38 and MDA-MB-468 cell lines stimulated with recombinant Wnt3a to identify a comprehensive list of Wnt target genes in breast cancer cells. A total of 19,738 transcripts were included on the array. The incubation of cells with Wnt3a for 6h, 12h, or 24h up-regulated (fold change > 1.3) the expression of 59, 64, and 22 genes, respectively in MDA-MB-468 cells (Fig 2A, S1 Dataset) and of 241, 385 and 362 genes, respectively in HCC38 cells (Fig 2B, S1 Dataset).

The difference in the number of up-regulated mRNAs between the two cell lines is consistent with the data obtained with the reporter assay showing that Wnt3a activates the Wnt pathway to a greater extent in HCC38 cells than in MDA-MB-468 cells (S1 Fig). In HCC38 cells, more genes were up-regulated at 12h and 24h after stimulation with Wnt3a than at 6h (Fig 2B, S1 Dataset). These genes may be “direct” or “secondary” Wnt target genes. However, one can postulate that the gene list found at the earliest time point (6h) is likely more enriched in “direct” Wnt target genes than those at later time points (12h, 24h). Therefore, we next focused on the genes up-regulated at 6h. Half of the target genes (30 out of 59) identified in MDA-MB-468 cells incubated for 6h with Wnt3a were also up-regulated in HCC38 cells after the same duration of treatment (Fig 2C, S2 Dataset). Among the 30 common genes, 23 were previously reported as Wnt target genes (Fig 2C, Table 1).

**Table 1:** Literature search for the 36 Wnt target genes (30 “up” and “6 down”) identified in both TNBC cell lines stimulated with Wnt3a for 6h.

Gene Symbol (official symbol)	ID	Wnt target gene	Role in Wnt/β-catenin signaling pathway	References
APCDD1	147495	yes	inhibitor	[36,40,54–58]
AXIN2	8313	yes	inhibitor	[35,37,56–63]
B3GNT2	10678		?	
BMP4	652	yes	?	[37,38,57,64]
BMP7	655	yes	inhibitor	[57,65]
C13orf15 (RGCC)	28984	yes	?	[58]
C21orf63 (EVA1C)	59271	yes	?	[58]
C2orf54	79919		?	
CYP24A1	1591	yes	?	[38]
DDIT4L	115265		?	
FAM171B	165215	yes	?	[58]
IGFL4	444882		?	
KRTAP3-1	83896	yes	?	[57]
LBH	81606	yes	?	[66]
LPAR1	1902		?	
MFSD2A	84879	yes	?	[58]
NKD1	85407	yes	inhibitor	[58,59,67–70]
PPFIBP1	8496	yes	?	[58]
PTGER2	5732	yes	?	[71]
RNF43	54894	yes	inhibitor	[37,57,58,72,73]
ROR1	4919	yes	?	[57]
SEMA3A	10371	yes	?	[35,37,56,59–63]
SLC39A10	57181	yes	?	[37]
SNCAIP	9627	yes	?	[58]
SNTB1	6641		?	
TFPI2	7980		?	
TMEM64	169200	yes	?	[58]
XKRX	402415	yes	?	[57]
ZNF703	80139	yes	?	[37,58]
ZNRF3	84133	yes	inhibitor	[37,57,58,73,74]
C10orf81 (PLEKHS1)	79949		?	
CLDN8	9073		?	
FBXO32	114907	yes	?	[40,58]
PCDH8	5100		?	
PPP1R3C	5507		?	
TLR1	7096		?	

As expected, the “universal” Wnt-induced genes *AXIN2* and *NKD1* [12] were up-regulated in both TNBC cell lines (Fig 2C, S2 Dataset). These two genes showed the highest fold change in HCC38 cells incubated with Wnt3a: 10.2 for *NKD1* (*P* = 2.2x10^-23^) and 9.86 for *AXIN2* (*P* = 6.4x10^-19^) (S2 Dataset). In addition to *AXIN2* [35,37,56–63] and *NKD1* [58,59,68–70], the 21 other previously reported Wnt target genes (Fig 2C, Table 1) that were up-regulated in both cell lines were: *APCDD1* [36,40,54–58], *BMP4* [37,38,57,64], *BMP7* [57], *C13orf15* [58], *C21orf63* [58], *CYP24A1* [38], *FAM171B* [58], *KRTAP3-1* [57], *LBH* [66], *MFSD2A* [58], *PPFIBP1* [58], *PTGER2* [71], *RNF43* [37,57,58,72,73], *ROR1* [57], *SEMA3A* [35,37,56,59–63], *SLC39A10* [37], *SNCAIP* [58], *TMEM64* [58], *XKRX* [57], *ZNF703* [37,58] and *ZNRF3* [37,57,58,73]. Therefore, we report seven novel Wnt target genes among the genes that were up-regulated in both cell lines: *B3GNT2*, *C2orf54*, *DDIT4L*, *IGFL4*, *LPAR1*, *SNTB1* and *TFPI2* (Table 1, Fig 2C). In addition to the 30 genes common to both cell lines at the 6h time point, which could be considered as specific to breast cancer cells, 29 and 211 Wnt target genes were specifically up-regulated in MDA-MB-468 and in HCC38 cells, respectively (Fig 2, S1 Dataset).

Altogether, this analysis revealed 270 genes that are potentially activated by Wnt3a at 6h; 133 (49%) were previously described as Wnt-induced genes and 137 are putative novel Wnt target genes (S3 Dataset).

### Genes repressed by Wnt3a in HCC38 and MDA-MB-468 triple-negative breast cancer cell lines

Although β-catenin is most commonly thought of as a transcriptional activator, several reports indicate that it may also be a transcriptional repressor [12,31,33,75]. Few studies have investigated this aspect of the canonical Wnt signaling pathway and it remains poorly understood. Genes down-regulated by Wnt signaling have been detected in human hepatoma cells displaying deregulated Wnt/β-catenin activities [39], in Wnt3a-stimulated human thymocytes, murine C3H10T1/2, NIH3T3 and PC12 cells [56,62,76], in human *CTNNB1*-mutated Wilms tumors [40], in intestinal tumors from *Apc(Min)* mice [77], and in a mouse mammary epithelial cell line stimulated with Wnt1 [34]. Some genes repressed by Wnt signaling are also mentioned in the “Wnt Home Page” website (http://www.stanford.edu/group/nusselab/cgi-bin/wnt/target_genes).

**Fig 3:**
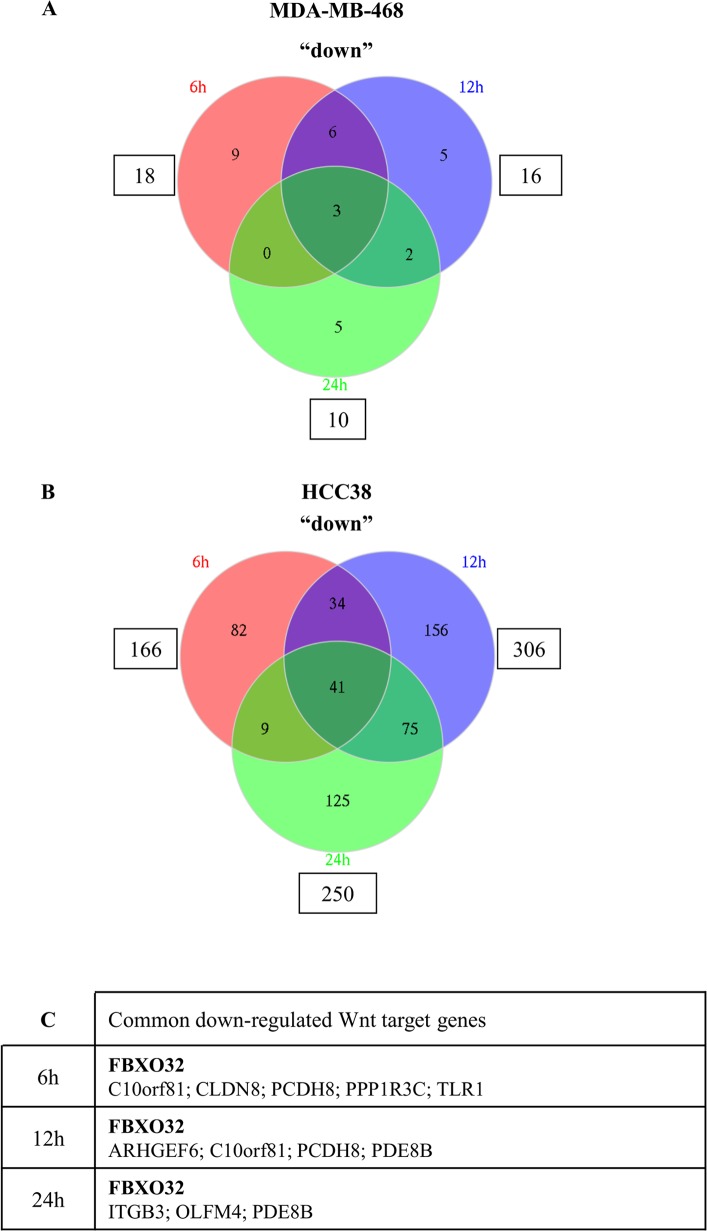
Wnt3a-dependent gene repression in MDA-MB-468 and HCC38 cells. Venn diagrams indicate the number of genes that were down-regulated in MDA-MB-468 (A) or HCC38 cells (B) following Wnt3a stimulation for 6, 12 or 24 hours. Genes found in common in both cell lines are listed in the table and known Wnt target genes are shown bold (C).

In agreement with these reports, we found that Wnt3a down-regulated the expression of several genes in TNBC cell lines. The incubation of cells with Wnt3a for 6h, 12h, or 24h down-regulated (fold change < 1.3) the expression of 18, 16, and 10 genes, respectively in MDA-MB-468 cells (Fig 3A, S1 Dataset) and 166, 306 and 250 genes, respectively in HCC38 cells (Fig 3B, S1 Dataset).

Wnt3a down-regulated the expression of more genes in HCC38 cells than in MDA-MB-468 cells, consistent with our previous observation that more genes are affected by Wnt3a in HCC38 cells than in MDA-MB-468 cells (Fig 2). One third (six out of 18) of the transcripts down-regulated in MDA-MB-468 cells incubated with Wnt3a for 6h were also down-regulated in HCC38 cells after the same treatment: *C10orf81*, *CLDN8*, *FBXO32*, *PCDH8*, *PPP1R3C* and *TLR1* (Fig 3C, S2 Dataset). Among them, *C10orf81*, *FBXO32* and *PCDH8* were also down-regulated in both cell lines after 12h of Wnt3a treatment (Fig 3C, S2 Dataset). The expression of *FBXO32* in stimulated cells after 24h of treatment was still lower than in resting control cells (Fig 3C, S2 Dataset). Except for *FBXO32* [40,58], none of the genes down-regulated in both TNBC cell lines have been previously identified as Wnt target genes (Table 1). In addition to these genes, 12 and 160 Wnt target genes were specifically down-regulated at 6h in MDA-MB-468 and in HCC38 cells, respectively (Fig 3, S1 Dataset).

Altogether, 178 potential targets were retrieved from our analysis; only 74 of them (42%) including *FBXO32* have been previously described as Wnt target genes (S3 Dataset). Therefore, our study revealed 104 putative novel target genes that are down-regulated in TNBC cells stimulated with Wnt3a (S3 Dataset).

### Validation of Wnt target genes in HCC38 and MDA-MB-468 triple-negative breast cancer cell lines

We sought to confirm that the Wnt targets identified by microarray analysis were deregulated in TNBC cells stimulated with Wnt3a; therefore, we examined the expression level of several genes by qRT-PCR. We selected early Wnt target genes (6h) found in both cell lines (“up”: *APCDD1*, *AXIN2*, *NKD1* and *DDIT4L*; “down”: *PCDH8* and *PPP1R3C*) (Fig 2C, Fig 3C, S2 Dataset). The magnitude of change in gene expression following Wnt3a treatment differed between qRT-PCR and microarray analysis, but the overall pattern was similar (Table 2 and Table 3, S2 and S3 Figs).

**Table 2:** qRT-PCR analysis of gene expression in MDA-MB-468 cells treated with Wnt3a.

		Wnt3a treatment							
		3h		6h		9h		12h	
	GENE SYMBOL	Fold change	+/- SD	Fold change	+/- SD	Fold change	+/- SD	Fold change	+/- SD
		*P* value		*P* value		*P* value		*P* value	
up	APCDD1	2.9 *	0.7	3.7 *	0.9	2.7 *	0.6	2.4 *	0.3
		<0.001		<0.001		<0.001		<0.001	
	AXIN2	6.6 *	1.5	**3.5** *	1.0	**2.7** *	0.6	**2.4** *	0.6
		<0.001		<0.001		<0.001		<0.001	
	DDIT4L	**14.0** *	5.7	**9.0** *	1.9	**7.0** *	1.3	**6.3** *	1.8
		<0.001		<0.001		<0.001		<0.001	
	NKD1	**1.5** *	0.6	**3.0** *	1.1	**1.9** *	0.5	**1.4** *	0.2
		0.024		<0.001		<0.001		0.002	
down	PCDH8	**1.2**	0.3	**-1.9** *	0.4	**-1.3**	0.4	**-1.8** *	0.4
		0.241		<0.001		0.152		<0.001	
	PPP1R3C	**-1.8** *	0.5	**-1.8** *	0.3	**-1.8** *	0.5	**-1.4** *	0.3
		<0.001		<0.001		<0.001		<0.001	

Fold change is relative to expression level in control cells (treated with PBS containing BSA). Results are expressed as the mean of three different experiments performed in triplicate. The significant *P* values are represented by a star (Student’s t test):

* *P*<0.05 (i.e. higher or lower expression *versus* control condition).

**Table 3:** qRT-PCR analysis of gene expression in HCC38 cells treated with Wnt3a.

		Wnt3a treatment							
		3h		6h		9h		12h	
	GENE SYMBOL	Fold change	+/- SD	Fold change	+/- SD	Fold change	+/- SD	Fold change	+/- SD
		*P* value		*P* value		*P* value		*P* value	
up	APCDD1	**2.2** *	0.9	**5.1** *	2.1	**4.4** *	1.7	**3.3** *	0.7
		0.002		<0.001		<0.001		<0.001	
	AXIN2	**17.3** *	4.4	**27.0** *	11.9	**16.7** *	4.6	**11.7** *	1.5
		<0.001		<0.001		<0.001		<0.001	
	DDIT4L	**7.5** *	2.3	**10.3** *	4.8	**7.8** *	4.4	**4.2** *	0.6
		<0.001		<0.001		<0.001		<0.001	
	NKD1	**4.6** *	1.4	**21.8** *	12.4	**20.6** *	12.6	**10.9** *	5.4
		<0.001		<0.001		<0.001		<0.001	
down	PCDH8	**-2.3** *	0.6	**-10.1** *	4.3	**-5.7** *	2.7	**-6.5** *	3.6
		<0.001		<0.001		<0.001		<0.001	
	PPP1R3C	**-4.1** *	0.6	**-4.8** *	1.5	**-4.3** *	1.5	**-3.4** *	0.9
		<0.001		<0.001		<0.001		<0.001	

Fold change is relative to expression level in control cells (treated with PBS containing BSA). Results are expressed as the mean of three different experiments performed in triplicate. The significant *P* values are represented by a star (Student’s t test):

* *P*<0.05 (i.e. higher or lower expression *versus* control condition).

The expression of *APCDD1*, *AXIN2*, *DDIT4L* and *NKD1* was up-regulated in MDA-MB-468 cells within the first 3-6h of Wnt3a treatment, and was subsequently down-regulated later on (Table 2). The expression of *PPP1R3C* was down-regulated at 3h of Wnt3a treatment and that of *PCDH8* was down-regulated at 6h of Wnt3a treatment (Table 2). We also carried out similar time-course experiments with Wnt3a in HCC38 cells (Table 3). The expression of *APCDD1*, *AXIN2*, *DDIT4L* and *NKD1* was up-regulated at 3h and reached a maximum level at 6h (Table 3). By contrast, the levels of *PPP1R3C* and *PCDH8* transcripts were lower in cells treated with Wnt3a for 3h than in resting cells, and reached their lowest levels at 6h (Table 3). The expression of *PPP1R3C* and *PCDH8* was more strongly down-regulated in HCC38 cells than in MDA-MB-468 cells, consistent with the microarray data (S2 Dataset, S2 and S3 Figs). Overall, qRT-PCR experiments on a selection of genes validated the microarray data and both analyses revealed a time-course of target gene expression similar to that reported by others [32,78]. The chronological order and duration of Wnt target gene activation/repression may be crucial for the controlled course of events downstream from the activated Wnt/β-catenin pathway in breast cancer. However, further investigation is required to explore this issue.

We sought to confirm that the regulation of the expression of the Wnt target genes was β-catenin-dependent; therefore, we transfected MDA-MB-468 cells with a construct encoding a transcriptionally active form of β-catenin, which carries a mutation in the GSK3β phosphorylation sites, rendering it refractory to the destruction complex. The experiment was not performed in HCC38 cells because these cells were harder to transfect. Luciferase assays showed that the expression of active β-catenin in MDA-MB-468 cells resulted in a very high level of Wnt activation in cells co-transfected with the WRE reporter plasmid (S4 Fig). The transcriptional activity of β-catenin was higher at 6h after transfection than in control cells, reached a peak at 12h and stayed constant until 24h.

Next, we measured the abundance β-catenin mRNA (to evaluate the efficiency of transfection, data not shown) and that of selected Wnt3a target genes by qRT-PCR in cells expressing the active, mutant form of β-catenin. The transfection of active β-catenin in MDA-MB-468 cells resulted in the up-regulation of *APCDD1*, *AXIN2*, *DDIT4L* and *NKD1* and the down-regulation of *PPP1R3C* expression (Table 4).

**Table 4:** qRT-PCR analysis of gene expression in MDA-MB-468 expressing the active, mutant form of β-catenin.

		expression of constitutively active β-catenin					
		12h		24h		48h	
	GENE SYMBOL	Fold change	+/- SD	Fold change	+/- SD	Fold change	+/- SD
		*P* value		*P* value		*P* value	
up	APCDD1	**1.5** *	0.4	**2.3** *	0.7	**3.5** *	0.6
		0.003		<0.001		<0.001	
	AXIN2	**2.2** *	0.6	**2.8** *	0.7	**4.4** *	1.1
		<0.001		<0.001		<0.001	
	DDIT4L	**2.3** *	0.7	**8.8** *	4.2	**11.4** *	4.8
		<0.001		<0.001		<0.001	
	NKD1	**1.1**	0.3	**1.6** *	0.4	**2.2** *	0.6
		0.557		<0.001		<0.001	
down	PCDH8	**-1.2**	0.3	**1.7** *	0.6	**-1.8**	1.1
		0.201		0.010		0.076	
	PPP1R3C	**-1.1**	0.3	**-1.6** *	0.8	**-1.6** *	0.3
		0.309		0.048		<0.001	

Fold change is relative to expression level in control cells (transfected with empty vector). Results are expressed as the mean of three different experiments performed in triplicate. The significant *P* values are represented by a star (Student’s t test):

* *P*<0.05 (i.e. higher or lower expression *versus* control condition).

Regarding *PCDH8*, the results were less clear as we noticed that the abundance of *PCDH8* mRNA in cells expressing activated β-catenin was higher at 24h, and tended to be lower 48h post-transfection (Table 4). This may be due to the cell line used for the experiment because PCDH8 was found to be highly down-regulated in HCC38 (max fold change = -10.1) and not to such an extent in MDA-MB-468 (max fold change = -1.9) upon Wnt3a stimulation (Table 2, Table 3). These results indicate that the expression of *APCDD1*, *AXIN2*, *DDIT4L*, *NKD1*, *PPP1R3C* and potentially *PCDH8* is regulated by Wnt3a through the activation of β-catenin.

These data confirmed that the analyzed genes are indeed Wnt/β-catenin target genes (some confirmation is required for *PCDH8* in another cell line) and that the genes identified in our microarray analysis at the earliest time point (6h) may therefore also be Wnt/β-catenin target genes. However, additional systematic experiments are required to confirm this hypothesis.

### Analysis of pathways deregulated in HCC38 and MDA-MB-468 triple-negative breast cancer cell lines upon Wnt3a treatment

The Wnt/β-catenin pathway activates but also represses the transcription of genes encoding products that may positively or negatively regulate the canonical Wnt pathway. Indeed, 34 out of 270 genes that were up-regulated in cells stimulated with Wnt3a for 6h, and 12 out of 178 genes that were down-regulated under the same conditions, encode known activators or inhibitors of this signaling pathway (S3 Dataset). Expression changes of these Wnt regulators are necessary to maintain Wnt signaling and to avoid the uncontrolled activation of the canonical pathway.

Although most Wnt target genes identified in both TNBC cell lines (24 out of 36, Table 1) have been previously described in other studies, their role in the Wnt/β-catenin pathway needs to be established. The exceptions to this are *APCDD1*, *AXIN2*, *BMP7*, *NKD1*, *RNF43* and *ZNRF3*, which are already known to regulate negatively the canonical pathway (S3 Dataset). Among the 12 potential novel Wnt target genes identified in HCC38 and MDA-MB-468 cells (Table 1, Fig 2C and Fig 3C), a link with the Wnt/β-catenin pathway was previously reported only for *DDIT4L*, *LPAR1* and *TLR1* [79–83].

REDD2 (product of the gene *DDIT4L*) activates the TSC (tuberous sclerosis complex) complex and negatively regulates mTOR signaling [79,84]. However, Wnt has also been shown to promote mTOR activity by preventing the activation of the TSC complex through two mechanisms: the inhibition of GSK3β, which is a known activator of the TSC complex, and the activation of the small GTPase Rac1 [80]. Additional experiments would be required to examine whether REDD2 induced by Wnt3a in TNBC cells attenuates mTOR activity that is initially stimulated by Wnt signaling (negative feedback loop).

In colon cancer cells, the binding of lysophosphatidic acid (LPA) to its receptors LPA_2_ and LPA_3_ but not to LPA_1_ (product of the gene *LPAR1*) activates the Wnt/β-catenin pathway [81]. Thus, it is possible that LPA_1_ plays a role in the aberrant activation of β-catenin signaling in TNBC cells. Further analyses are needed to confirm this hypothesis.

Only one paper mentions a link between Wnt and TLR1, reporting that Wnt3a suppresses pro-inflammatory responses to TLR1/2 ligands in dendritic cells [82].

We used KEGG analysis to examine enriched pathways among the genes positively or negatively regulated by Wnt3a in each cell line to explore the effect of Wnt signaling on cellular functions (S4 Dataset).

We focused mainly on HCC38 cells because the number of deregulated genes was very different between the two TNBC cell lines and higher in HCC38 cells (Fig 2 and Fig 3). Wnt target genes that were up-regulated in HCC38 cells stimulated for 6h with Wnt3a were associated with the TGFβ, Wnt, p53 and Hedgehog signaling pathways, whereas the down-regulated Wnt target genes were associated with calcium and mTOR signaling pathways (S4 Dataset). Some of these pathways (e.g., TGFβ and p53 pathways) were still deregulated even after 12h or 24h of treatment. Many Wnt target genes play a role in Wnt regulation (S3 Dataset); therefore, we expected to find the Wnt signaling pathway among the affected pathways. Interestingly, the pathways affected in both HCC38 cells and MDA-MB-468 cells (S4 Dataset) control proliferation (e.g., Hedgehog, Jak-STAT, p53 and Wnt signaling), apoptosis/autophagy (e.g., p53 and mTOR signaling) and inflammation/immune responses (e.g., TGFβ and toll-like receptor signaling, and cytokine-cytokine receptor interactions). These results are not surprising given the well documented crosstalk between Wnt signaling and these pathways [80,85–92]. Moreover, Hedgehog, TGFβ, Jak-STAT and toll-like receptor signaling pathways are deregulated in breast cancer and play a role in its development [93–96]. Altogether, these data may provide insight into the effect of aberrant Wnt/β-catenin signaling on biological processes involved in the development of TNBC/BLBC.

### Comparison of our list of Wnt target genes identified in HCC38 and MDA-MB-468 triple-negative breast cancer cell lines with previously published lists

We compared our list of Wnt target genes with previously published lists (activated and repressed genes were compared separately) (S5 Dataset) [34,35,37–40,56–63,97,98] to determine whether the Wnt target genes that we identified were specific to breast cancer cells.

First, we compared our lists of Wnt target genes to those reported in NIH3T3 fibroblasts [56,97] and in C3H10T1/2 mesenchymal cells [35,37,56,59–63] stimulated with Wnt3a. Of the Wnt target genes identified in C3H10T1/2 cells, four out of the 21 up-regulated genes (*AHR*, *AXIN2*, *SEMA3A* and *TGFB3*), but none of the 19 repressed genes responded to Wnt3a in TNBC cells [35,37,56,59–63]. Eleven genes that were up-regulated in response to Wnt3a in fibroblasts (*AHR*, *APCDD1*, *ARL4C*, *AXIN2*, *FAS*, *FZD7*, *GADD45G*, *IRS1*, *KLF5*, *TGFB3* and *WNT11*) and three genes that were down-regulated (*CITED2*, *PDGFRA* and *PTGFR*) were also found in our study [56,97].

Second, the comparison was performed with Wnt target genes identified in cancer cells other than breast tumors. Nine of 76 Wnt target genes up-regulated in ovarian endometrioid adenocarcinoma (*BMP4*, *CCND1*, *CYP24A1*, *FGF9*, *IRS1*, *MSX2*, *PCSK6*, *SEMA3C* and *SFN*) were also identified in our study [38]. Thirteen out of 208 Wnt target genes overexpressed in colorectal tumors [37] were also up-regulated by Wnt signaling in TNBC cells: *AXIN2*, *CDC25A*, *KIAA1199*, *LEF1*, *MET*, *MYB*, *PHLDA1*, *SLC19A2*, *SLC25A19*, *SLC39A10*, *SOX4*, *ZNF703* and *ZNRF3*. Several other studies have also investigated the Wnt signature in the intestine. Herbst et al. identified deregulated genes in DLD1 and SW480 cells [58] and compared the potential target genes with data published for LS174T cells [57]. Among the 193 genes commonly regulated by β-catenin in the three colorectal tumor cell lines (61 “up” and 132 “down”) [58], 12 were also overexpressed (*APCDD1*, *AXIN2*, *C10orf2*, *CDC25A*, *DEPDC7*, *EDAR*, *FGF9*, *KIAA1199*, *NAV3*, *RNF43*, *VSNL1* and *ZNRF3*) and one was repressed (*ELF3*) in response to Wnt3a in our TNBC cells. *FBXO32*, which was identified in both TNBC cell lines, was down-regulated in DLD1, not in SW480 cells [58]. Among the 122 target genes identified as overexpressed in Wilms tumors, only *APCDD1* was common to our study [40]. *FUT8* was the only Wnt target gene among 54 up-regulated genes in hepatoma cells that was also up-regulated in response to Wnt3a in TNBC cells [39]. *AXIN2* and *IRS1* were the only Wnt target genes in breast cancer cells that were also identified in hepatocellular carcinoma cells [63].

Finally, we compared our Wnt target genes to those found in normal and cancerous mammary cells. One study examined gene expression in the mouse mammary epithelial cell line C57MG in response to Wnt3a [35]; 16% of Wnt3a-induced genes (*AXIN2*, *AHR*, *CCND1*, *EFNB2*, *IRS1* and *KLF5*) and 8% of the repressed genes (*ANGPT1*) found in this study were also identified in our TNBC cells. Another study examined gene expression in C57MG cells stimulated by Wnt1, another canonical ligand [34]. Interestingly, *IRF7* was the only common gene that responded to both Wnt3a and Wnt1 in these cells (see list of “down” genes) [34,35]. We found that *EGR1*, *FAS* and *SLC7A2* were up-regulated and *DIO2*, *ETS1* and *VEGFA*, were down-regulated in TNBC in response to Wnt3a, consistent with reports in Wnt1-treated C57MG cells [34,35]. Among the 248 target genes identified in normal murine mammary gland epithelial cells treated with Wnt3a, seven were common to our study (“up”: *AHR*, *AXIN2*, *CCND1*, *GADD45G* and *HS3ST1*; “down”: *MMP13* and *TNFAIP2*) [98].

Overall, these observations show that very few Wnt target genes identified in breast cancer cells were common to other tissues; thus, only a handful can be considered as ‘universal’ target genes e.g. *APCDD1*, *AXIN2* and *IRS1* (S5 Dataset). Unexpectedly, *NKD1*, another well-known Wnt target gene, was reported in only one [58] out of the 13 selected studies (S5 Dataset). Although they are derived from the same tissue, the TNBC cell lines HCC38 and MDA-MB-468 display strong differences regarding the number and the identity of Wnt target genes. Similar observations were made in the colorectal cancer lines DLD1 and SW480 depleted in β-catenin [58] (S5 Dataset). In addition, C57MG mammary epithelial cells treated with Wnt1 [34] or Wnt3a [35], two canonical ligands from two different classes, display different gene expression profiles (S5 Dataset), suggesting that a particular Wnt ligand affects the expression of a specific set of Wnt target genes.

Altogether, these data confirm that Wnt target genes are diverse, tissue-, cell line- and treatment-specific. Therefore, we believe it is necessary to perform similar analyses in many different breast cancer cell lines (triple negative/basal-like and luminal) after stimulation with various Wnt ligands to obtain a more complete picture of Wnt target genes in this tissue.

### Wnt target genes are enriched in human breast tumor samples

We next sought to examine, in samples of human TNBC, the expression level of the Wnt target genes identified in HCC38 stimulated with Wnt3a for 6, 12 or 24h, to investigate whether the expression of these genes correlates with potential Wnt activation in these tumors. To achieve sufficient statistical power, the analyses were performed with the data obtained with HCC38 cells, and not with those obtained with MDA-MB-468 cells, because few genes were found deregulated in MDA-MB-468 cells (see Materials and Methods section for details). The comparative analyses between cell line and tumors were performed with transcriptomic data previously obtained with our cohort composed of 130 human breast cancer tumors [41,42].

**Fig 4:**
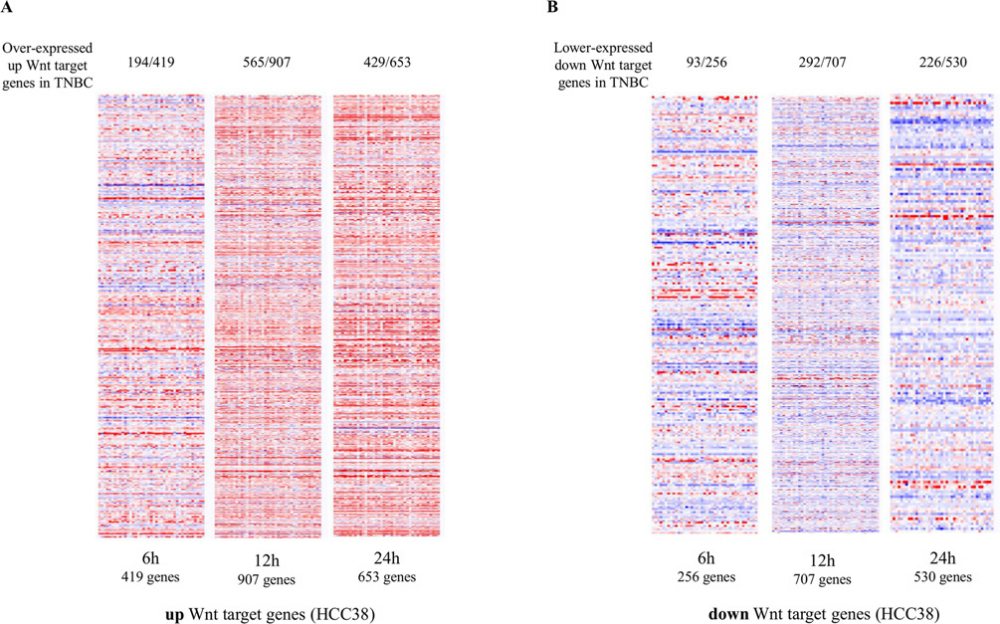
Wnt target genes identified in Wnt3a-stimulated HCC38 cells and their expression in TNBC samples. We identified Wnt target genes that were differentially expressed in HCC38 cells stimulated with Wnt3a (6h, 12h or 24h), and we examined the expression level of up-regulated (A) and down-regulated (B) Wnt target genes in TNBC samples. We restricted the study to the 11262 genes present on both cell line and tumor arrays. We applied a FDR (False Discovery Rate) cut-off of 0.05 and a log-2 fold change threshold of 0. The numbers of up- regulated (A) and down- regulated (B) genes taken into account are indicated at the bottom for each time point (6h, 12h and 24h). The numbers of the Wnt target genes that were more strongly (A) or more poorly (B) expressed in TNBC than in LA samples are indicated on top of the figure. Rows: genes; columns: tumor samples. Red: more strongly expressed genes; blue: more poorly expressed genes. Genes are ordered by gene Entrez ID. The names of the genes are indicated in S8 Fig.

Many of the Wnt target genes that were up-regulated in Wnt3a-stimulated HCC38 cells were more strongly expressed in TNBC than in Luminal A (LA) samples (Fig 4A, Table 5). We refer to this overexpression as “enrichment” in the following analysis (down-regulated genes can similarly be defined as enriched). Genes identified in HCC38 cells stimulated with Wnt3a for 6h, 12h or 24h were enriched in TNBC samples, with the most significant enrichment corresponding to genes identified at 12h and 24h (Fig 4A, Table 5). Similarly, Wnt target genes were enriched among the genes more strongly expressed in TNBC than in HER2+ or Luminal B (LB) samples (Table 5). In addition, a significant proportion of the Wnt target genes down-regulated in HCC38 cells were more poorly expressed (and hence enriched) in TNBC than in LA samples (Fig 4B, S1 Table), or than in HER2+ or LB samples (S1 Table).

**Table 5:** Wnt target genes up-regulated in Wnt3a-stimulated HCC38 cells and their enrichment in human breast cancer samples.

		genes up-regulated in HCC38 cells			
	Wnt3a	6h	12h	24h	6h & 24h
	gene nb	419	907	653	133
TNBC vs LA	3497	194 *3*.*5x10* ^*-11*^	565 *3*.*8x10* ^*-91*^	429 *1*.*1x10* ^*-78*^	72 *4*.*0x10* ^*-8*^
HER2+ vs LA	2110	110 *1*.*2x10* ^*-4*^	358 *1*.*2x10* ^*-52*^	288 *1*.*8x10* ^*-53*^	35 *0*.*033*
LB vs LA	1587	65 *0*.*39*	250 *9*.*4x10* ^*-29*^	230 *8*.*7x10* ^*-45*^	25 *0*.*15*
TNBC vs HER2+	2242	142 *8*.*9x10* ^*-12*^	429 *7*.*8x10* ^*-85*^	334 *5*.*8x10* ^*-76*^	55 *1*.*7x10* ^*-8*^
TNBC vs LB	2775	171 *1*.*1x10* ^*-13*^	506 *4*.*6x10* ^*-98*^	383 *1*.*6x10* ^*-81*^	66 *4*.*0x10* ^*-10*^
HER2+ vs LB	581	36 *0*.*0031*	93 *6*.*7x10* ^*-11*^	74 *1*.*0x10* ^*-10*^	13 *0*.*027*

We assessed the significance of the overlap between the genes that were up-regulated in Wnt3a-stimulated HCC38 (6h, 12h, 24h, and those up-regulated at both 6h and 24h) and those that were strongly expressed in tumors (TNBC, HER2+, LB and LA) (gene nb). We restricted our study to the 11262 genes present on both arrays (HCC38 dataset, tumor dataset). The number of the Wnt target genes that were more strongly expressed is indicated for each tumor comparison. The associated *P* value is also shown underneath (*in Italics*). The significance of the overlap between two lists was assessed with the Fisher exact test. As an example, 194 of the 419 Wnt target genes up-regulated in Wnt3a-stimulated HCC38 cells (6h) were more strongly expressed in TNBC than in LA samples (194 of the 3497 genes more strongly expressed genes in TNBC than in LA samples) (*P* value = 3.5x10^-11^).

We also obtained similar results for the comparison of HER2+ and LB with LA samples, although the findings were less significant (Table 5, S1 Table). Wnt target genes that were up-regulated in cells stimulated with Wnt3a for 6h were more strongly expressed and enriched in HER2+ than in LA samples, but were expressed to a similar extent in LB and LA samples (Table 5). Those identified at 12h and 24h of Wnt3a stimulation were more strongly expressed and enriched in both HER2+ and LB samples than in LA samples (Table 5). Wnt target genes down-regulated in Wnt3a-stimulated cells were also more poorly expressed and enriched in HER2+ and LB samples than in LA samples (S1 Table).

In conclusion, this analysis revealed that the Wnt target genes that were up- or down-regulated in Wnt3a-stimulated HCC38 cells were highly enriched in TNBC and, to a lesser extent, in HER2+ and LB tumors. This may indicate that the Wnt pathway is activated in these three breast cancer subtypes, with the highest activation in TNBC. However, it is well established that the activation of the Wnt/β-catenin pathway induces cell proliferation, in agreement with our KEGG pathway analysis (S4 Dataset). Therefore, long exposure (24h) of cells to Wnt3a will also lead to the activation or repression of genes linked to proliferation. Breast cancer subtypes proliferate at different rates: TNBC are the most proliferative tumors, followed by HER2+, LB and LA tumors (for an example, see the expression profile of several markers of proliferation in tumors, S5 Fig). Thus, it is possible that in breast cancer subtypes, the enrichment of Wnt target genes identified in cell lines stimulated with Wnt3a for 12h or 24h reflects proliferation, which may, or may not, be related to Wnt activation. However, it is impossible to distinguish Wnt activation from proliferation, because both are closely linked.

**Fig 5:**
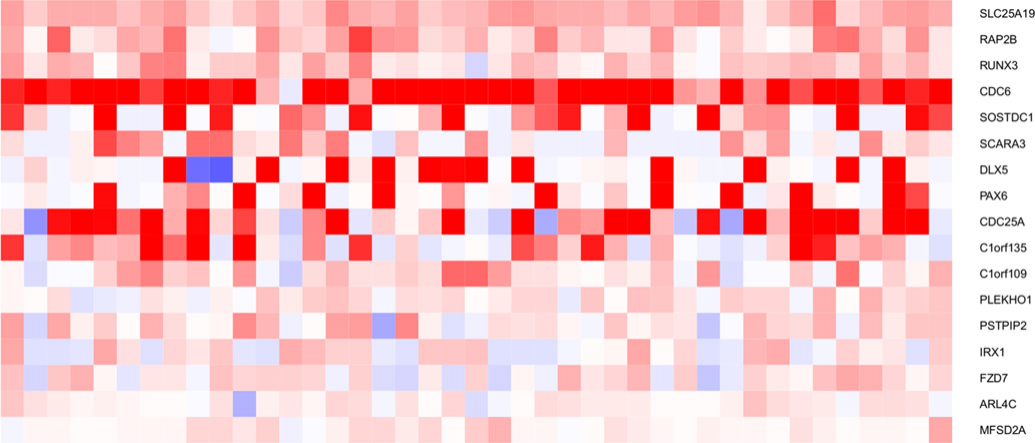
Seventeen Wnt target genes up-regulated in HCC38 cells are strongly expressed in TNBC tumors and may reflect chronic activation of the Wnt signaling pathway. To identify potentially up-regulated Wnt target genes that could reflect the chronic activation of the Wnt pathway in human cancer, we selected the Wnt target genes that were up-regulated at both the earliest (6h) and the latest (24h) time points after the stimulation of HCC38 cells with Wnt3a. Of 133 genes up-regulated in Wnt3a-stimulated HCC38 cells at both time points, 72 genes were more strongly expressed in TNBC than in LA tumors (see S6 Fig). When we restricted our analysis to the most strongly deregulated genes (with a fold change > 1.3), 17 genes out of 72 genes were retained. The genes are ordered by their *P* value in the t-test for the TNBC subgroup. Rows: genes; columns: tumor samples. Red: more strongly expressed genes; blue: more poorly expressed genes.

Finally, we hypothesize that the Wnt target genes found in TNBC cell lines as soon as 6h after Wnt3a stimulation, that continue to be differentially expressed at late time points (24h) may be deregulated during the chronic activation of the Wnt pathway, such as that occurring in tumors. We focused on Wnt target genes that were up-regulated in Wnt3a-stimulated HCC38 cells because only a small number of genes were common to both time points in MDA-MB-468 cells: 133 and 13 genes were shared between the 6h and 24h time points in HCC38 and MDA-MB-468 cells, respectively (selected from a gene list restricted to those present on both the cell line and the tumor arrays; see Material and Methods for details). Of these 133 genes, 54% (72 genes) were more strongly expressed and enriched (*P* = 4.0x10^-8^) in TNBC than in LA tumors (Table 5, S6 Fig). These Wnt target genes were also enriched in TNBC vs. HER2+ (*P* = 1.7x10^-8^) or LB tumors (*P* = 4.0x10^-10^) (Table 5). Interestingly, these genes were also significantly enriched in HER2+ tumors (*P* = 0.033), albeit to a lesser extent than in TNBC, but were not enriched in LB tumors (*P* = 0.15) (Table 5). The high expression of these 72 genes may reflect an activation of the Wnt pathway in TNBC samples. When we restricted our analysis to the most strongly deregulated genes (with a fold change > 1.3), 17 genes out of 72 genes were retained (Fig 5), including 11 genes previously identified as Wnt target genes: *SLC25A19*, *CDC6*, *CDC25A*, *C1orf109*, *C1orf135*, *PLEKHO1*, *PAX6*, *PSTPIP2*, *MFSD2A*, *FZD7*, *ARL4C* (S3 Dataset). Among those genes, we identified the Frizzled 7 receptor (FZD7) (Fig 5). Interestingly, high levels of FZD7 reported in TNBCs have been previously proposed to drive Wnt activation in these tumors [23]. Of note, the expression profiles of most of these 17 genes appear to not be related to proliferation (S7 Fig). In conclusion, the strong expression of these 17 Wnt target genes may reflect the activation of the canonical Wnt pathway in a more chronic situation, in particular in TNBCs.

## Supporting Information

S1 Dataset Microarray data of Wnt target genes (WTG) identified in TNBC cell lines stimulated with Wnt3a. (XLS)

S2 Dataset Microarray data of Wnt target genes (WTG) identified in both TNBC cell lines treated with Wnt3a. (XLS)

S3 Dataset Literature search to identify relevant information about Wnt target genes identified in TNBC cell lines stimulated with Wnt3a for 6h. (XLSX)

S4 Dataset Pathway enrichment according to KEGG classification of genes positively or negatively regulated by Wnt3a in TNBC cells. (XLS)

S5 Dataset The expression profile of Wnt target genes in different cell/tissue contexts—Comparison of our list with previously published lists. (XLSX)

S1 Fig Transcriptional activity of β-catenin/Tcf in MDA-MB-468 and HCC38 cells treated with Wnt3a. MDA-MB-468 (A) or HCC38 cells (B) were transiently transfected with WRE or MRE and pRL-TK plasmids. The transcriptional activity of β-catenin/Tcf was evaluated after Wnt3a or vehicle stimulation (3, 6, 9 or 12 hours). The error bars show the standard deviation of the mean and asterisks indicate a significant *P* value in Student’s t test (* *P*<0.05, i.e. higher luciferase activity *versus* control condition).
(TIF)

S2 Fig Microarray data of Wnt target genes up-regulated (A) and down-regulated (B) in MDA-MB-468 cells treated with Wnt3a. Gene expression was evaluated in TNBC cells in the presence (red dots) or the absence (blue dots) of Wnt3a ligand and results are expressed as log2 values. The fold change between treated and control cells is indicated when significant (*P* < 0.05). ns: not significant.
(TIF)

S3 Fig Microarray data of Wnt target genes up-regulated (A) and down-regulated (B) in HCC38 cells treated with Wnt3a. Gene expression was evaluated in TNBC cells in the presence (red dots) or the absence (blue dots) of Wnt3a ligand and results are expressed as log2 values. The fold change between treated and control cells is indicated when significant (*P* < 0.05). ns: not significant.
(TIF)

S4 Fig Transcriptional activity of β-catenin/Tcf in MDA-MB-468 cells expressing an active, mutant form of β-catenin. Cells were co-transfected with pRK5-SK-β-cateninΔGSK or pRK5-SK and WRE or MRE and pRL-TK plasmids. The transcriptional activity of β-catenin/Tcf was evaluated 6, 12, and 24 hours after transfection. The error bars show the standard deviation of the mean and asterisks indicate a significant *P* value in Student’s t test (* *P*<0.05, i.e. higher luciferase activity *versus* control condition).
(TIF)

S5 Fig Expression profile of proliferation markers in human breast cancer samples. mRNA expression of 4 markers of proliferation are shown in TNBC, HER2+, luminal B (LB), luminal A (LA) samples as well as in normal breast tissues (norm). RNA quantifications were logarithmic (log2) transformed and illustrated by boxplots.
(TIF)

S6 Fig A list of the 72 Wnt target genes up-regulated in Wnt3a-stimulated HCC38 cells and overexpressed in TNBC tumors that may reflect chronic activation of the Wnt signaling pathway. To identify potentially up-regulated Wnt target genes that could reflect the chronic activation of the Wnt pathway in human cancer, we selected the Wnt target genes that were up-regulated at both the earliest time point (6h) and the latest time point (24h) after the stimulation of HCC38 cells with Wnt3a. Of the 133 genes up-regulated in HCC38 cells at both time points, 72 were more strongly expressed in TNBC than in LA tumors. The genes are ordered by their *P* value in the t-test for the TNBC subgroup. Rows: genes; columns: tumor samples. Red: more strongly expressed genes; blue: more poorly expressed genes.
(TIF)

S7 Fig Expression profile of the 17 Wnt target genes that may reflect chronic activation of the Wnt signaling pathway in breast cancer samples. The abundance of mRNA of the 17 Wnt target genes is shown for TNBC, HER2+, luminal B (LB), luminal A (LA) samples as well as normal breast tissues (norm). The values were log2 transformed and are illustrated by boxplots.
(TIF)

S8 Fig Wnt target genes identified in Wnt3a-stimulated HCC38 cells and their expression in TNBC samples. Identical data that are in Fig 4 but with the names of the genes indicated.
(PDF)

S1 Table Wnt target genes down-regulated in Wnt3a-stimulated HCC38 cells and their enrichment in human breast cancer samples. (DOCX)
