# Supplementary figures and images for: Transcriptome Analysis of Wnt3a-Treated Triple-Negative Breast Cancer Cells
Source: PLoS One. 2015 Apr 7;10(4):e0122333. doi: 10.1371/journal.pone.0122333 (PMC4388387; doi:10.1371/journal.pone.0122333)

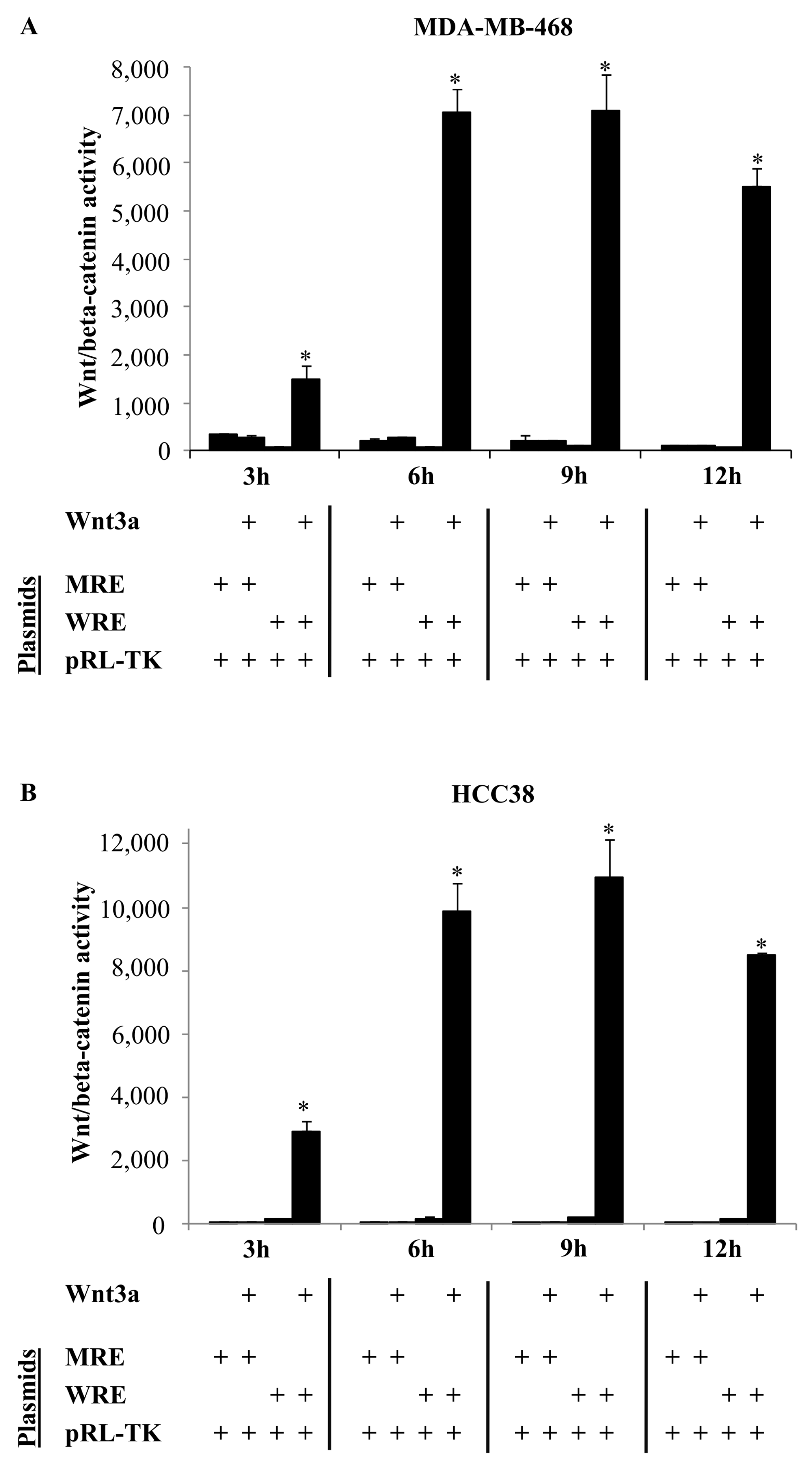

Supplement: S1 Fig — MDA-MB-468 (A) or HCC38 cells (B) were transiently transfected with WRE or MRE and pRL-TK plasmids. The transcriptional activity of β-catenin/Tcf was evaluated after Wnt3a or vehicle stimulation (3, 6, 9 or 12 hours). The error bars show the standard deviation of the mean and asterisks indicate a significant P value in Student’s t test (* P<0.05, i.e. higher luciferase activity versus control condition). (TIF) [file pone.0122333.s006.tif]

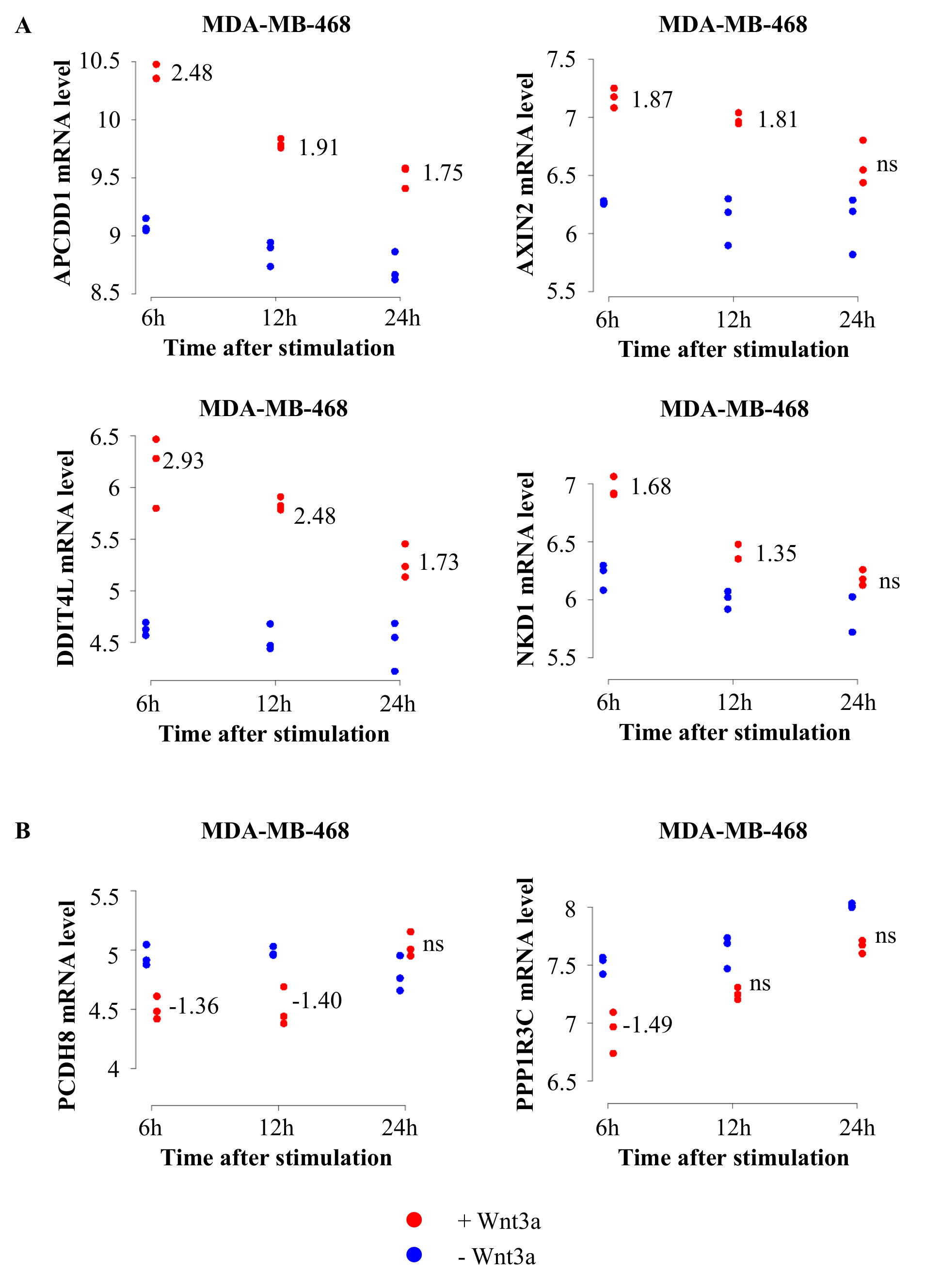

Supplement: S2 Fig — Gene expression was evaluated in TNBC cells in the presence (red dots) or the absence (blue dots) of Wnt3a ligand and results are expressed as log2 values. The fold change between treated and control cells is indicated when significant (P < 0.05). ns: not significant. (TIF) [file pone.0122333.s007.tif]

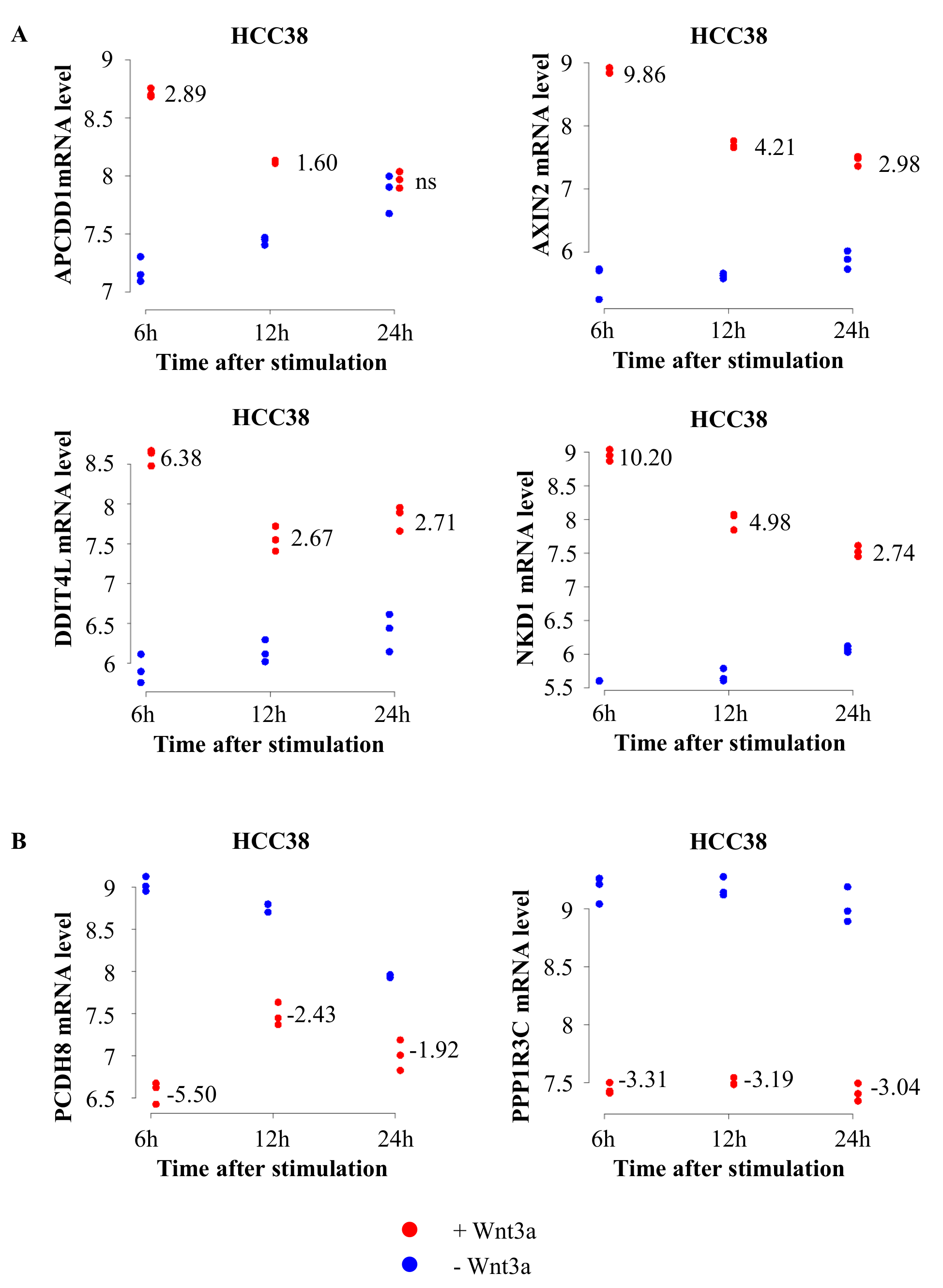

Supplement: S3 Fig — Gene expression was evaluated in TNBC cells in the presence (red dots) or the absence (blue dots) of Wnt3a ligand and results are expressed as log2 values. The fold change between treated and control cells is indicated when significant (P < 0.05). ns: not significant. (TIF) [file pone.0122333.s008.tif]

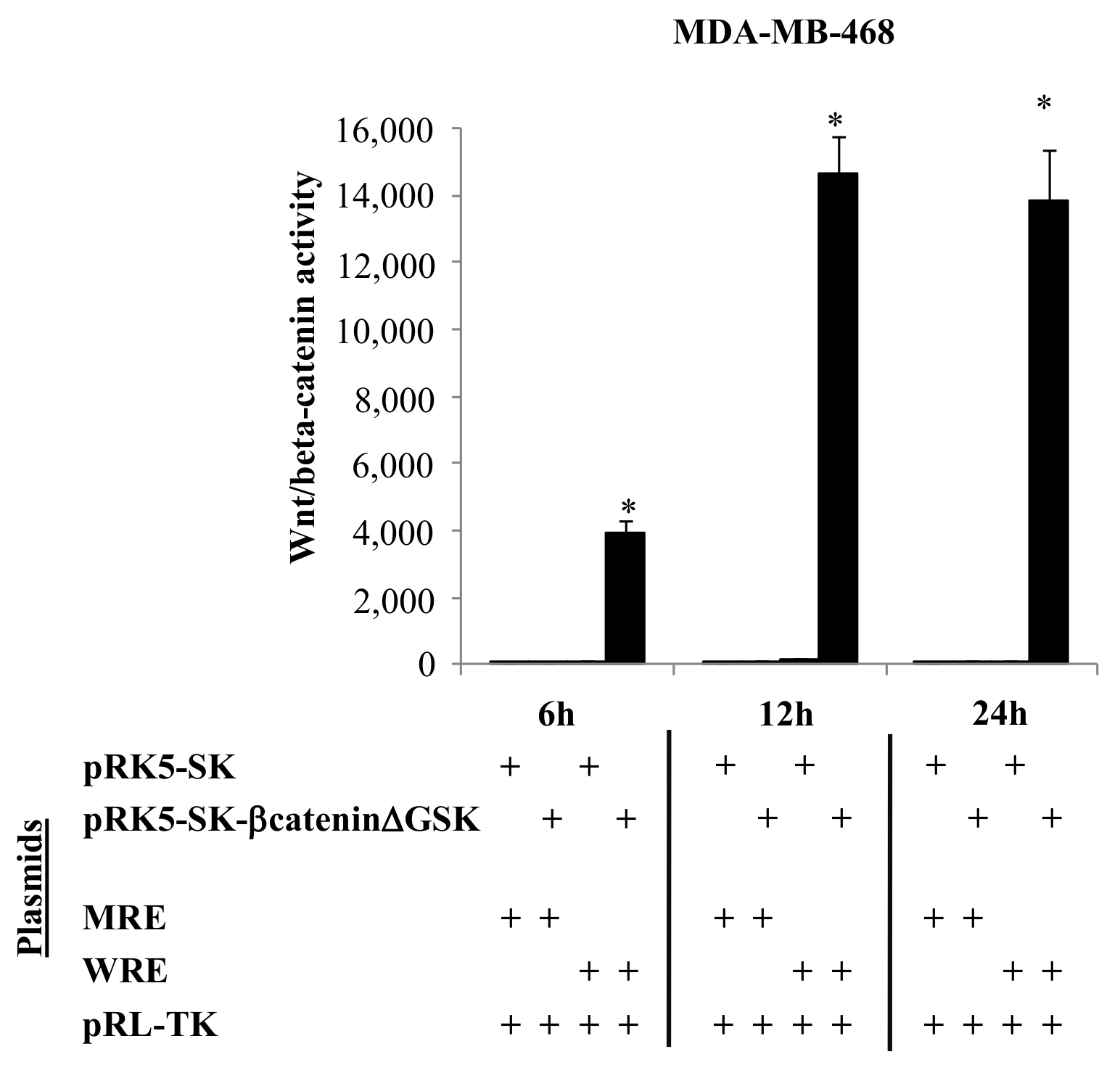

Supplement: S4 Fig — Cells were co-transfected with pRK5-SK-β-cateninΔGSK or pRK5-SK and WRE or MRE and pRL-TK plasmids. The transcriptional activity of β-catenin/Tcf was evaluated 6, 12, and 24 hours after transfection. The error bars show the standard deviation of the mean and asterisks indicate a significant P value in Student’s t test (* P<0.05, i.e. higher luciferase activity versus control condition). (TIF) [file pone.0122333.s009.tif]

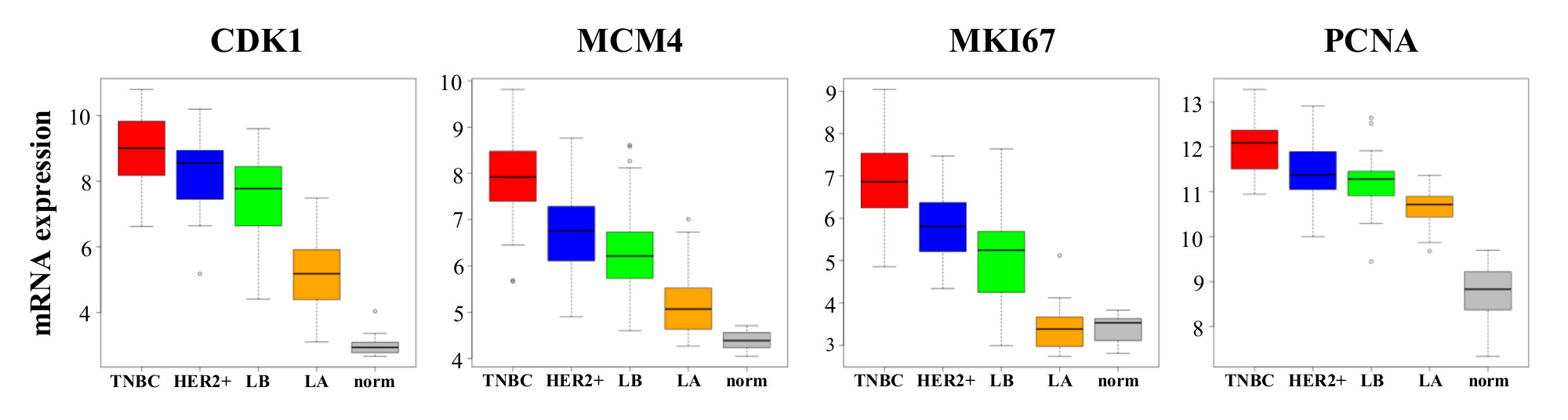

Supplement: S5 Fig — mRNA expression of 4 markers of proliferation are shown in TNBC, HER2+, luminal B (LB), luminal A (LA) samples as well as in normal breast tissues (norm). RNA quantifications were logarithmic (log2) transformed and illustrated by boxplots. (TIF) [file pone.0122333.s010.tif]

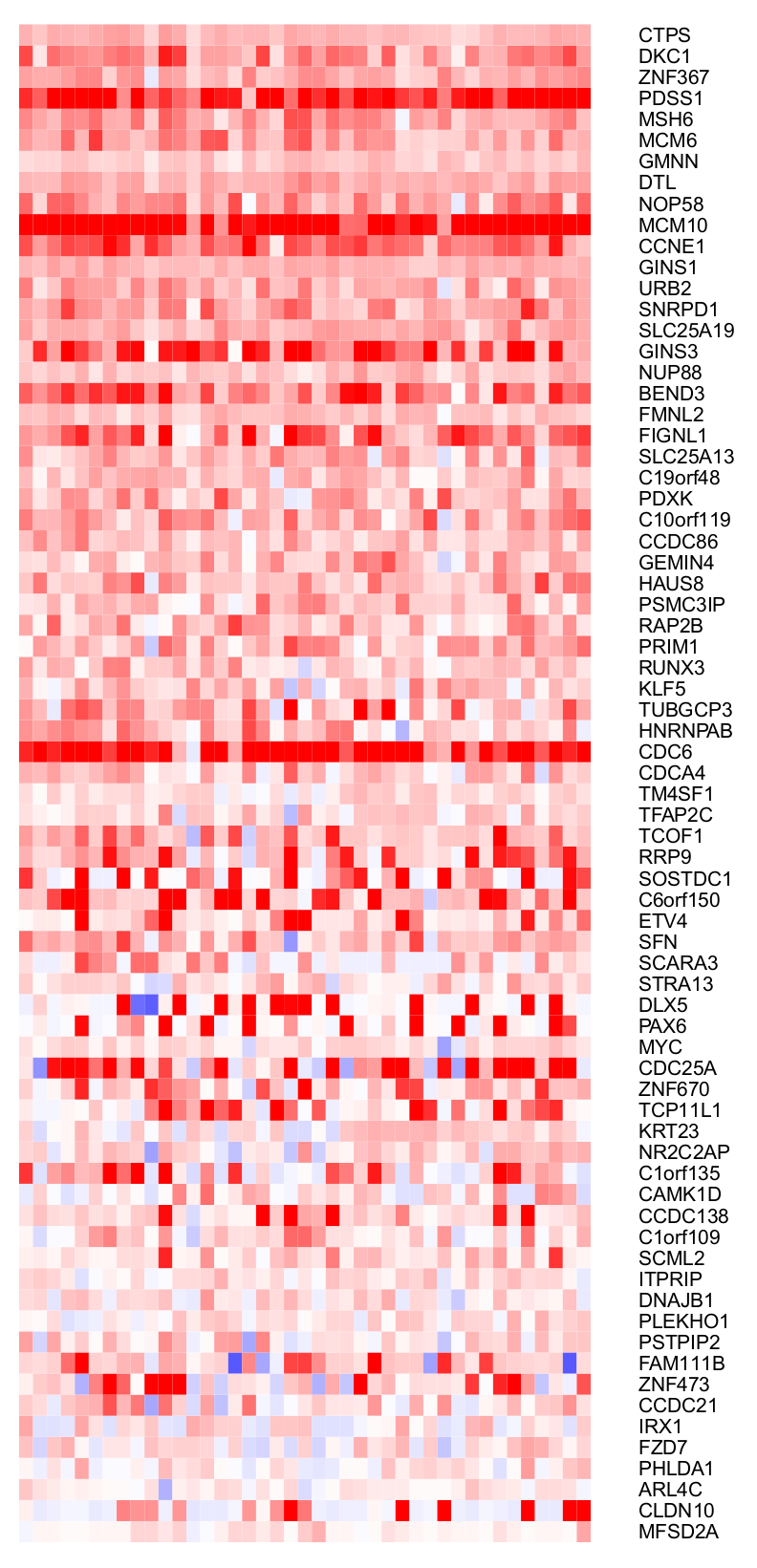

Supplement: S6 Fig — To identify potentially up-regulated Wnt target genes that could reflect the chronic activation of the Wnt pathway in human cancer, we selected the Wnt target genes that were up-regulated at both the earliest time point (6h) and the latest time point (24h) after the stimulation of HCC38 cells with Wnt3a. Of the 133 genes up-regulated in HCC38 cells at both time points, 72 were more strongly expressed in TNBC than in LA tumors. The genes are ordered by their P value in the t-test for the TNBC subgroup. Rows: genes; columns: tumor samples. Red: more strongly expressed genes; blue: more poorly expressed genes. (TIF) [file pone.0122333.s011.tif]

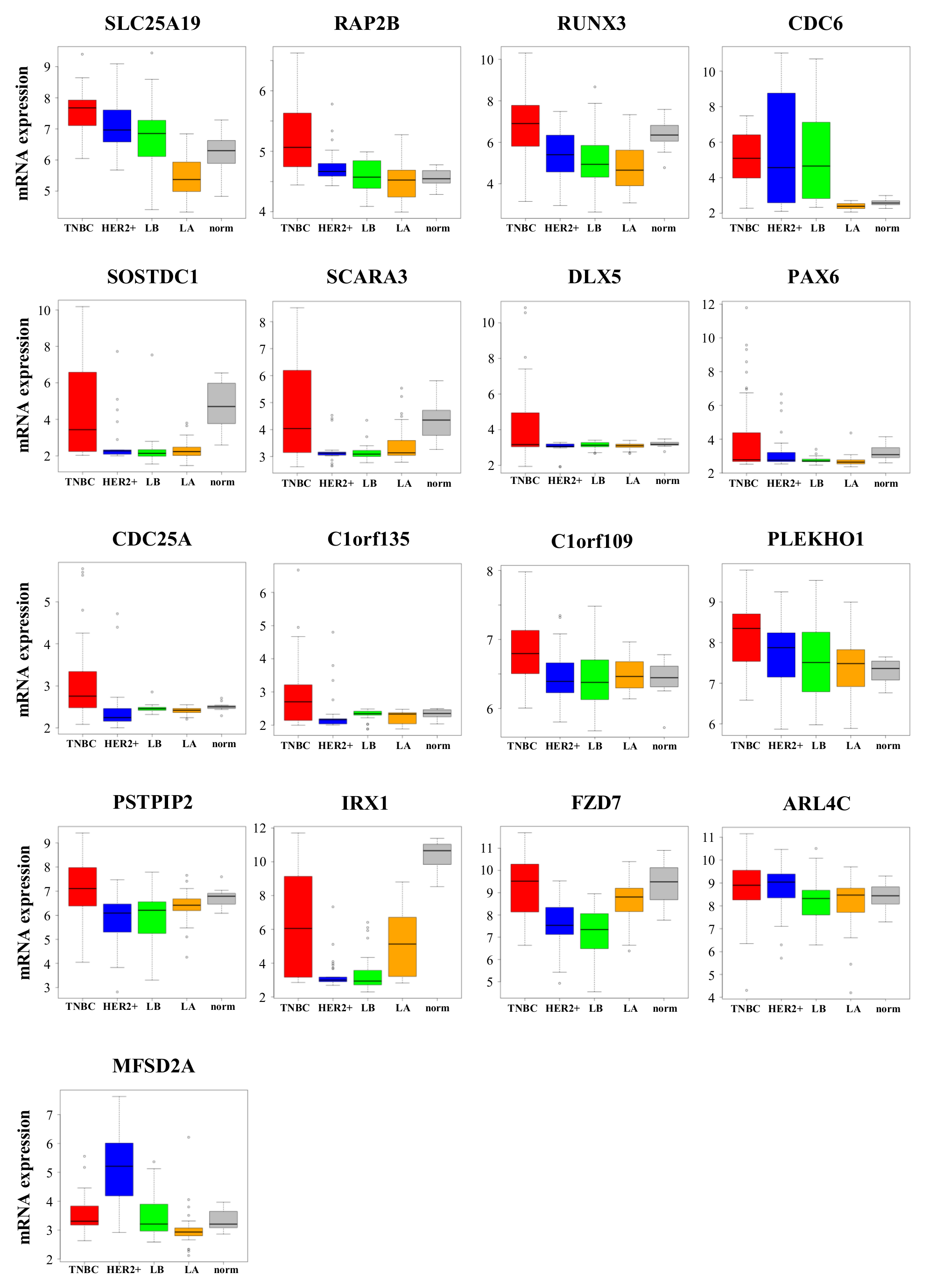

Supplement: S7 Fig — The abundance of mRNA of the 17 Wnt target genes is shown for TNBC, HER2+, luminal B (LB), luminal A (LA) samples as well as normal breast tissues (norm). The values were log2 transformed and are illustrated by boxplots. (TIF) [file pone.0122333.s012.tif]
