# Supplementary material for: Transcriptome Analysis of Wnt3a-Treated Triple-Negative Breast Cancer Cells
Source: PLoS One. 2015 Apr 7;10(4):e0122333. doi: 10.1371/journal.pone.0122333 (PMC4388387; doi:10.1371/journal.pone.0122333)

## up−6h

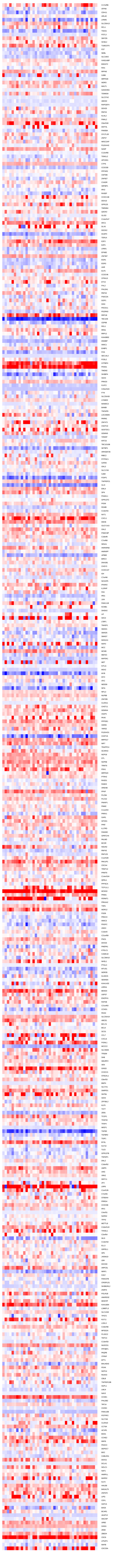

## up−12h

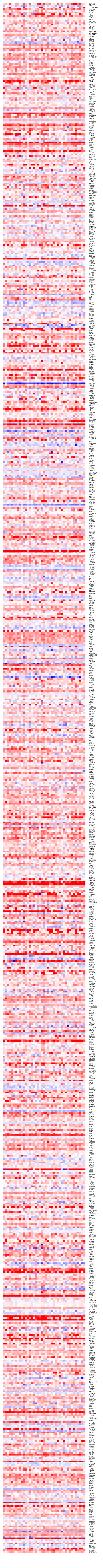

## up−24h

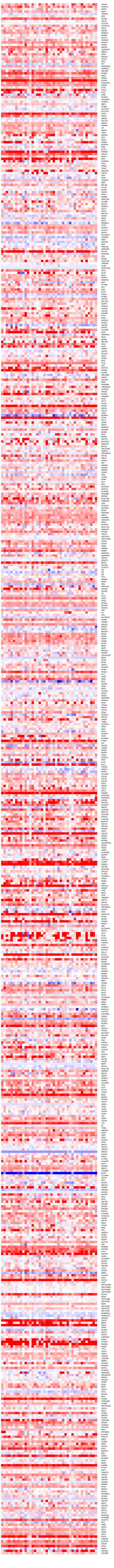

**down−6h**

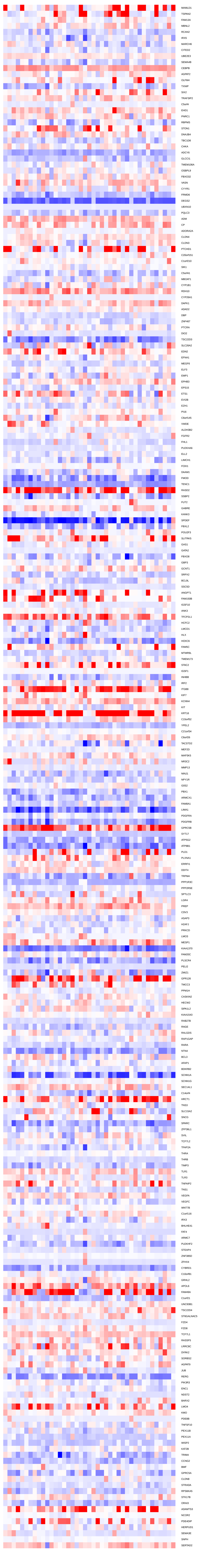

## down−12h

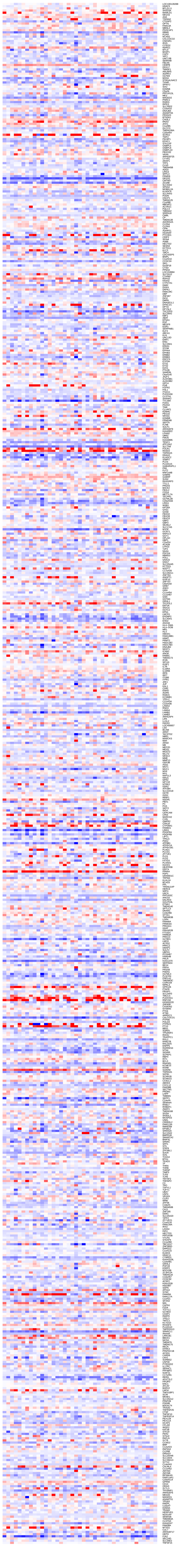

**down−24h**

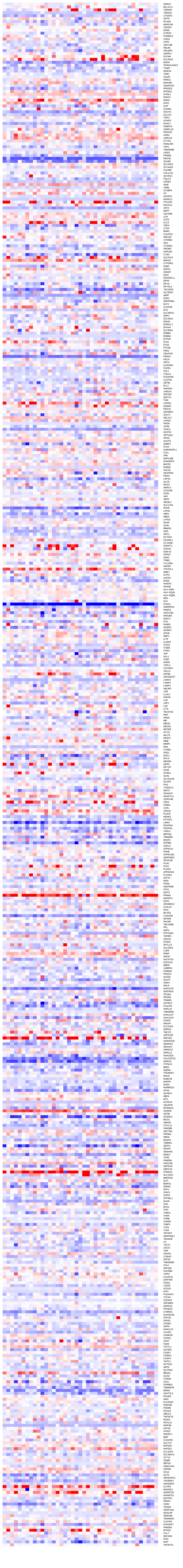

Supplement: S8 Fig — Identical data that are in Fig 4 but with the names of the genes indicated. (PDF) [file pone.0122333.s013.pdf]
