# Supplementary material for: Transcriptome Analysis of Wnt3a-Treated Triple-Negative Breast Cancer Cells
Source: PLoS One. 2015 Apr 7;10(4):e0122333. doi: 10.1371/journal.pone.0122333 (PMC4388387; doi:10.1371/journal.pone.0122333)
Supplement: S1 Table — (DOCX) [file pone.0122333.s014.docx]

**S1_Table. Wnt target genes down-regulated in Wnt3a-stimulated HCC38 cells and their enrichment in human breast cancer samples**

|  |  | genes down-regulated in HCC38 cells | | |
| --- | --- | --- | --- | --- |
|  | Wnt3a | 6h | 12h | 24h |
|  | gene nb | 256 | 707 | 530 |
| TNBC vs LA | 2971 | 93 *4.2x10^-4^* | 292 *5.3x10^-19^* | 226  *1.2x10^-16^* |
| HER2+ vs LA | 2001 | 59 *0.031* | 190 *6.0x10^-10^* | 135  *5.2x10^-6^* |
| LB vs LA | 1184 | 47 *1.2x10^-4^* | 116 *6.7x10^-7^* | 102  *7.0x10^-10^* |
| TNBC vs HER2+ | 1865 | 72 *2.4x10^-6^* | 196  *1.2x10^-14^* | 164  *4.2x10^-17^* |
| TNBC vs LB | 2725 | 71 *0.18* | 259  *2.3x10^-14^* | 193  *1.2x10^-10^* |
| HER2+ vs LB | 581 | 22 *0.021* | 36  *1.00* | 29  *0.69* |

We assessed the significance of the overlap between the genes that were down-regulated in Wnt3a-stimulated HCC38 (6h, 12h and 24h) and those that were poorly expressed in tumors (TNBC, HER2+, LB and LA) (gene nb). We restricted our study to the 11262 genes present on both arrays (HCC38 dataset, tumor dataset). The number of the Wnt target genes that were more poorly expressed is indicated for each tumor comparison. The associated *P* value is also shown underneath (*in Italics*). The significance of the overlap between two lists was assessed with the Fisher exact test. As an example, 93 of the 256 Wnt target genes down-regulated in Wnt3a-stimulated HCC38 cells (6h) were more poorly expressed in TNBC than in LA samples (93 of the 2971 genes more poorly expressed genes in TNBC than in LA samples) (*P* value=4.2x10^-4^).
